# Rap2B drives tumorigenesis and progression of colorectal cancer through intestinal cytoskeleton remodeling

**DOI:** 10.1038/s41419-025-07627-8

**Published:** 2025-04-13

**Authors:** Jiehui Di, Zhongjun Zhao, Mingyi Xia, Keyu Gao, Keli Chai, Bao Zhu, Wanping Sun, Yanping Zhang, Junnian Zheng, Yong Liu

**Affiliations:** 1 Cancer Institute, Xuzhou Medical University, 209 Tongshan Road, Xuzhou, Jiangsu 221004 China; 2 Center of Clinical Oncology, The Affiliated Hospital of Xuzhou Medical University, 99 West Huaihai Road, Xuzhou, Jiangsu 221002 China; 3 Jiangsu Center for the Collaboration and Innovation of Cancer Biotherapy, Xuzhou Medical University, 209 Tongshan Road, Xuzhou, Jiangsu 221004 China

**Keywords:** Prognostic markers, Colorectal cancer, Oncogenes

## Abstract

Ras family protein plays a key role in transducing signals involved in cytoskeletal remodeling and cell adhesion, which are particularly important in the development of colorectal cancer (CRC). While Rap2B, a member of the Ras superfamily, has been linked to cancer malignancy in vitro, its exact role in tumorigenesis remains unclear. In this study, we demonstrated that intestine-specific knockout of Rap2B suppresses the initiation and progression of CRC. Mechanistically, Rap2B interacts with plectin and enhances its expression, which in turn inhibits plectin-mediated F-actin assembly. Deletion of Rap2B resulted in a remodeling of the intestinal cytoskeleton, leading to reduced tumorigenesis and diminished metastatic potential. Clinically, there is a positive correlation between the expression levels of Rap2B and plectin in human CRC tissues, and higher levels of Rap2B and plectin predicting poorer clinical outcome in CRC patients. These findings underscore a critical role of Rap2B in CRC progression and highlight its potential as a therapeutic target.

## Introduction

The cytoskeleton is essential for maintaining intestinal homeostasis by establishing and preserving cellular polarity, which is vital for the secretion, digestion, and absorption functions of intestinal epithelial cells [1–3]. Disruption of the intestinal epithelial barrier through alterations in the actin cytoskeleton is linked to colitis, which may further progress to colorectal cancer (CRC) [4–6].

Ras family proteins play a crucial role in signal transduction, particularly in processes such as cell adhesion and cytoskeletal remodeling[7, 8]. Rap proteins belong to the Ras superfamily and share approximately 50% sequence identity with classical RAS proteins. In humans, five distinct genes of the Rap family have been identified: Rap1A, 1B, 2 A, 2B, and 2 C. These Rap proteins are primarily involved in cell adhesion, motility, and actin cytoskeleton dynamics [9–11]. Recent studies have highlighted Rap2 as a central integrator of cytoskeletal signals for Hippo signaling [12–15]. These findings have spurred further interest in elucidating the precise functions and regulatory mechanisms of Rap2 GTPase, especially its interactions with the cytoskeleton.

Rap2B, a member of the Rap2 family, was initially discovered in the early 1990s by screening of a platelet cDNA library [16]. Rap2B possesses three well-defined domains characteristic of Ras proteins: an effector domain (amino acids 32-40), a nucleotide binding domain, and a C-terminal CAAX motif (C, cysteine; A, aliphatic residue; X, any amino acid) [17, 18]. Notably, the amino acid sequence of Rap2B is highly conserved, suggesting that this protein may play a significant role throughout evolution [19]. Previous studies, including our own, have shown that Rap2B is highly expressed in various human cancers and acts as an oncogene in multiple tumor types in vitro [20–26]. However, the in vivo oncological function and the mechanisms behind it remain uncharacterized, which significantly limits the potential for developing anti-tumor therapies targeting this gene.

Plectin, a scaffolding protein which bridges cytoskeleton networks, has emerged as a significant driver of malignant hallmarks in various cancers [27]. Plectin are widely expressed and abundant in many tissues, playing a crucial role in many cellular processes associated with tumorigenesis, including cell adhesion, migration, and signal transduction [22]. Notably, plectin knockout can enhance F-actin assembly and subsequently inhibit cell movement [28, 29].

In this study, we demonstrated that intestine-specific knockout of Rap2B suppresses the initiation and progression of colitis and CRC. Mechanistically, Rap2B interacts with plectin and enhances its expression, which in turn inhibits plectin-mediated F-actin assembly. Importantly, we observed a positive correlation between the expression levels of Rap2B and plectin in CRC tissues, with higher levels of Rap2B and plectin predicting increased metastatic potential and poorer prognostic outcomes in CRC patients. Together, our findings highlight the Rap2B-plectin-F-actin axis as a novel regulatory pathway that plays a critical role in the initiation and progression of CRC.

## Results

### Rap2B positively correlates with increased metastasis and poor prognosis in CRC patients

**Fig. 1:**
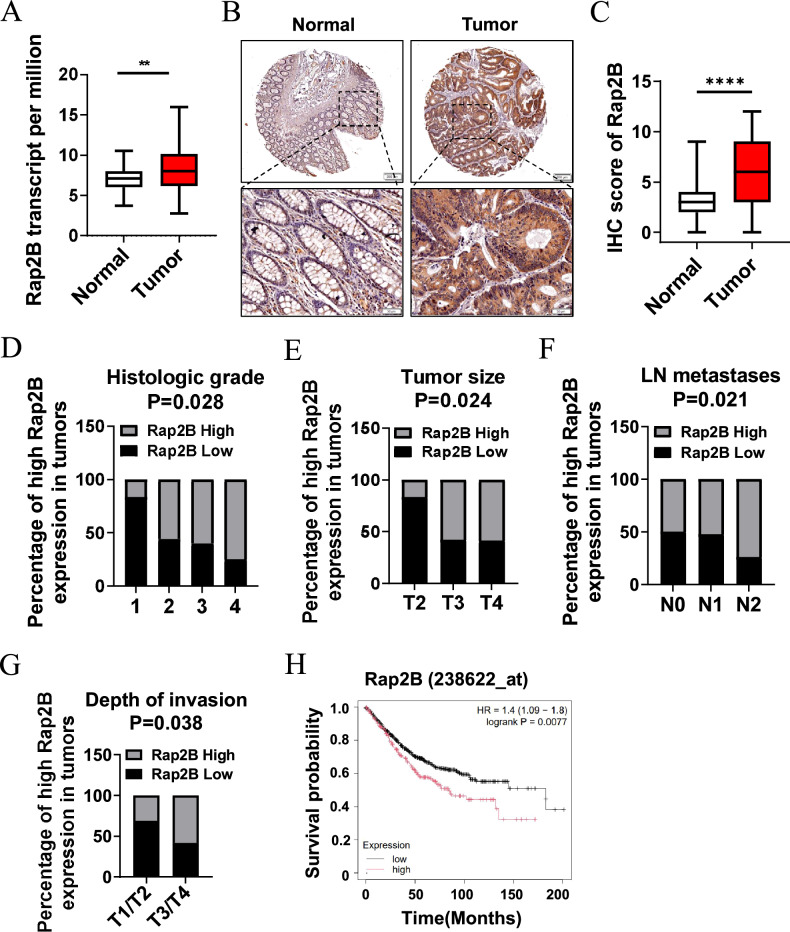
Rap2B positively correlates with increased metastasis and poor prognosis in CRC patients. **A** Analysis of Rap2B expression in colon cancer in TCGA database. **B**, **C** Representative images and IHC intensity analysis using Rap2B antibody in 179 human colon cancer and 161 adjacent normal colon tissue. The percentages of high Rap2B expression levels correlated with histologic grades (**D**), tumor sizes (**E**), LN metastasis (**F**) and distant metastasis (**G**) were examined by χ2 test. **H** High Rap2B expression were correlated with overall survival in 814 colon cancer patients (High Rap2B patients 214, Low Rap2B patients 600) (P = 0.0077, log-rank test). Data are presented as mean ± SEM. *P < 0.05, **P < 0.01, ***P < 0.001 by 2-tailed unpaired t test.

We first assessed Rap2B expression in 286 CRC samples and 41 non-tumor samples from The Cancer Genome Atlas (TCGA). The results showed that Rap2B was significantly upregulated in CRC tissues compared to non-tumor tissues (Fig. 1A). Immunohistochemistry (IHC) on tissue microarray (TMA) further confirmed that Rap2B protein expression was significantly increased in CRC compared to adjacent normal colon tissues (Fig. 1B, C). Next, we evaluated the relationship between Rap2B levels and clinicopathological characteristics in human CRC specimens. Our analysis revealed a positive correlation between high Rap2B levels and prognostic factors such as histological grade, tumor stage, lymph node (LN) metastasis, and depth of invasion (Fig. 1D-G, Table 1). Notably, data from the Kaplan Meier plotter database indicated that high Rap2B expression is associated with poor overall survival in CRC patients (Fig. 1H), suggesting a significant role for Rap2B in CRC progression.

**Table 1:** Rap2B staining and clinicopathological characteristics of 179 CRC patients.

	Rap2B Staining			
Variables	Low (%)	High (%)	Total	*P* value
**Gender**				
Male	39 (42.86)	52 (57.14)	91	0.421
Female	40 (45.45)	48 (54.55)	88	
**Age**				
<57	23 (53.49)	20 (46.51)	43	0.105
≥57	55 (41.04)	79 (58.96)	134	
**Tumor size**				
T2	10 (83.33)	2 (16.67)	12	0.024
T3	34 (41.98)	47 (58.02)	81	
T4	35 (41.18)	42 (49.41)	85	
**Lymph node metastasis**				
N0	54 (50.00)	54 (50.00)	108	0.021
N1	21 (47.73)	23 (52.27)	44	
N2	7 (25.93)	20 (74.07)	27	
**Distant metastasis**				
Negative	77 (45.03)	94 (54.97)	171	0.23
Positive	2 (25.00)	6 (75.00)	8	
**Depth of invasion**				
T1/T2	11 (68.75)	5 (31.25)	16	0.038
T3/T4	68 (41.72)	95 (58.28)	163	
**Clinical staging**				
1	10 (83.33)	2 (16.67)	12	0.028
2	41 (44.09)	52 (55.91)	93	
3	26 (40.00)	39 (60.00)	65	
4	2 (25.00)	6 (75.00)	8	
**Pathologic typing**				
Adenocarcinoma	53 (42.40)	72 (57.60)	125	0.576
Mucinous adenocarcinoma	24 (48.98)	25 (51.02)	49	
Signet ring cell carcinoma	2 (40.00)	3 (60.00)	5	

### IEC-specific Rap2B deletion inhibits colorectal cancer tumorigenesis

**Fig. 2:**
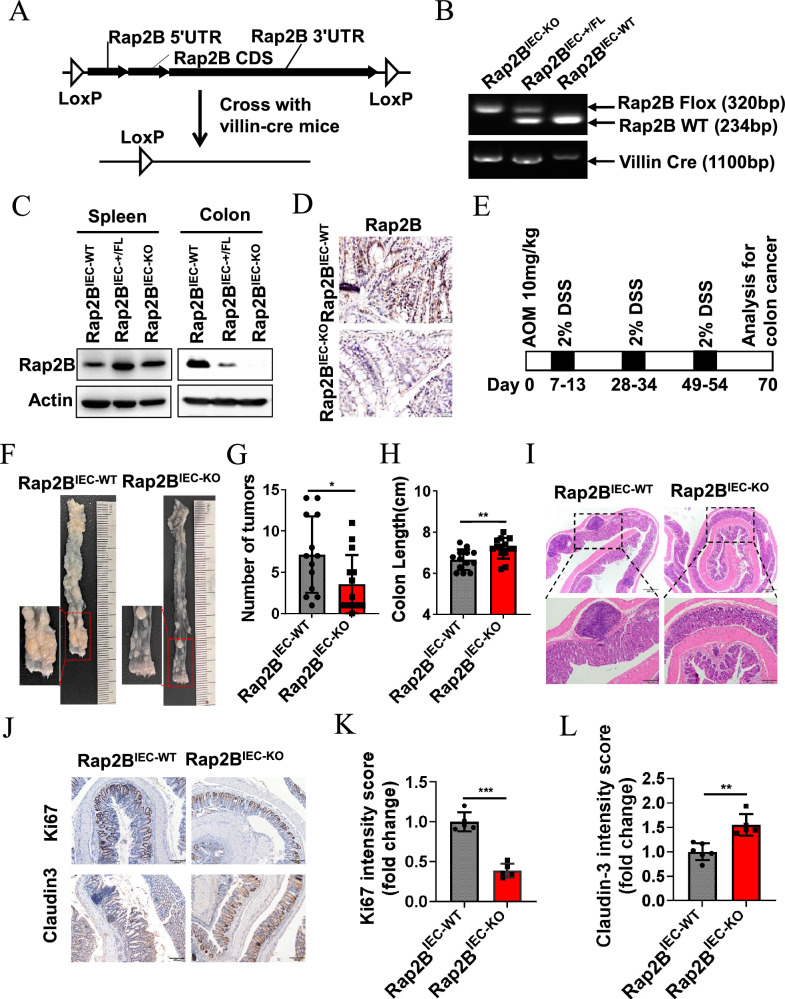
IEC-specific Rap2B deletion inhibits colorectal cancer tumorigenesis. **A** Schematic representation of strategies used to generate *Rap2B*^flox/flox^ and *Rap2B*^IEC-KO^ mice. **B** Genotyping PCR analysis using tail DNAs from *Rap2B*^IEC-WT^, *Rap2B*^IEC-+/FL^ and *Rap2B*^IEC-KO^ showing the flox and WT alleles of Rap2B and the Cre transgene. **C**, **D** Representative immunoblot analysis and IHC staining confirming loss of Rap2B expression in the colon tissue of *Rap2B*^IEC-KO^ mice. **E** Schematic of the experimental strategy to induce CRC model. **F** Representative images of colons from *Rap2B*^IEC-WT^ and *Rap2B*^IEC-KO^ mice with CRC induction model. Zoomed images of boxed region are shown at the left. **G**, **H** Quantification of tumor incidence per colon and colon length of *Rap2B*^IEC-WT^ and *Rap2B*^IEC-KO^ mice with CRC induction model (n = 13). **I** Representative H&E analysis of the colons from *Rap2B*^IEC-WT^ and *Rap2B*^IEC-KO^ mice with CRC induction model. **J**–**L** Representative micrographs of immunochemistry staining for Ki-67 and Claudin-3 in tumors from *Rap2B*^IEC-WT^ and *Rap2B*^IEC-KO^ mice with CRC induction model (n = 5). Data in J–H, K and L are presented as the mean ± SEM. *P < 0.05, **P < 0.01, ***P < 0.001 by 2-tailed unpaired t test.

To investigate the in vivo role of Rap2B in regulating intestinal tumorigenesis, we generated intestinal epithelial-specific Rap2B knockout mice (*Rap2B*^IEC-KO^ mice) by crossing *Rap2B*-flox mice with *Villin*-Cre transgenic mice (Fig. 2A–D). *Rap2B*^IEC-KO^ mice gained weight at the same rate as wild-type (*Rap2B*^IEC-WT^) mice and exhibited similar colon lengths, along with normal crypt-villus structures (Fig. S1A-E). Additionally, intestinal epithelial cells displayed comparable proliferation capacity, as indicated by Ki67 staining (Fig. S1F-G).

We then investigated the role of Rap2B in the colitis-associated CRC model. In this model, *Rap2B*^IEC-WT^ mice and *Rap2B*^IEC-KO^ mice were injected with the colonotropic mutagen Azoxymethane (AOM), followed by Dextran Sodium Sulfate (DSS) treatment [30] (Fig. 2E). Notably, while AOM/DSS treatment induced colon tumors in both genotypes, the treated *Rap2B*^IEC-KO^ mice exhibited a reduced number of colon tumors (Fig. 2F, G), longer colon lengths (Fig. 2H), and improved histological outcomes with more regular intestinal epithelial structures (Fig. 2I) compared to *Rap2B*^IEC-WT^ mice. Furthermore, the expression of tight junction proteins Claudin3 increased and the cell proliferation marker Ki67 decreased in the colorectal tissues of *Rap2B*^IEC-KO^ mice compared to the *Rap2B*^IEC-WT^ control mice, indicating that the deletion of Rap2B contributes to maintaining the integrity of the intestinal epithelial barrier and prevents aberrant epithelial cell proliferation (Fig. 2J–L). Taken together, these findings demonstrate that Rap2B has a pivotal role in the development of colon cancer.

### IEC-specific Rap2B deletion inhibits the progression of colitis

**Fig. 3:**
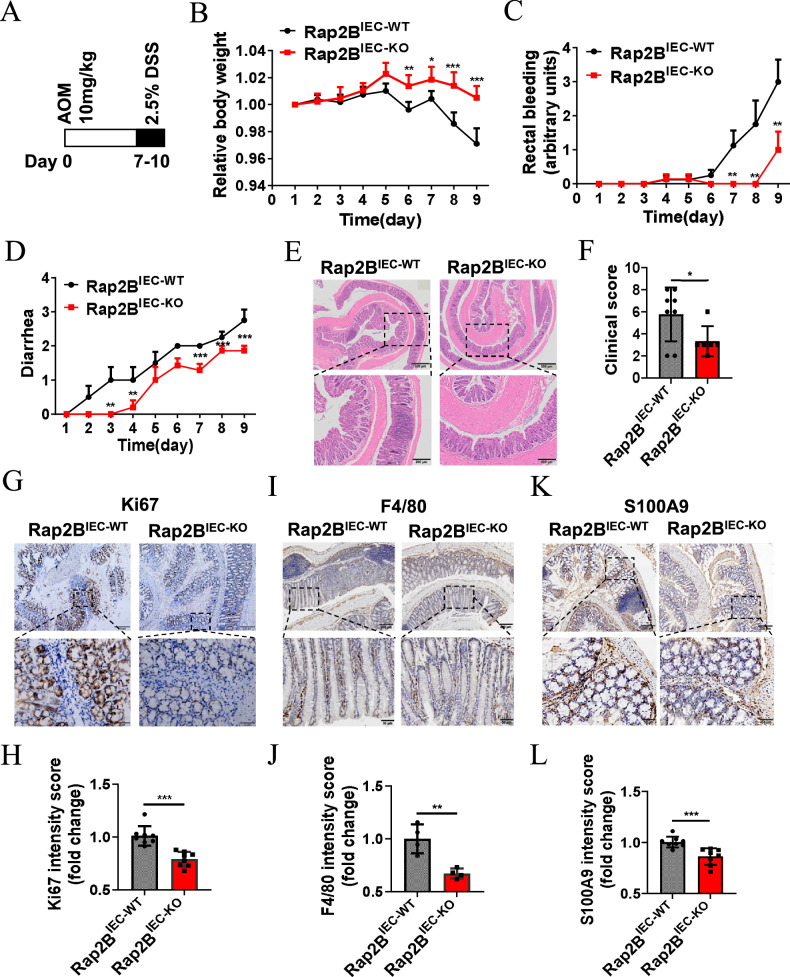
IEC-specific Rap2B deletion inhibits the progression of colitis. **A** Schematic of the experimental strategy to induce colitis model. **B–D** Quantification of the relative body weight, rectal bleeding and diarrhea of *Rap2B*^IEC-WT^ and *Rap2B*^IEC-KO^ mice with colitis model induction (n = 8). **E**, **F** Representative H&E analysis and clinical score of the colons from *Rap2B*^IEC-WT^ (n = 8) and *Rap2B*^IEC-KO^ (n = 6) mice with colitis model induction. **G**, **H** Representative micrographs of immunochemistry staining for Ki-67 and quantitative analysis in tumors from *Rap2B*^IEC-WT^ and *Rap2B*^IEC-KO^ mice with colitis model induction (n = 4; 2 images/mice). **I**, **J** Representative micrographs of immunochemistry staining for F4/80 and quantitative analysis in tumors from *Rap2B*^IEC-WT^ and *Rap2B*^IEC-KO^ mice with colitis model induction (n = 4). **K**, **L** Representative micrographs of immunochemistry staining for S100A9 and quantitative analysis in tumors from *Rap2B*^IEC-WT^ and *Rap2B*^IEC-KO^ mice with colitis model induction (n = 4; 2 images/mice). Data in **J**, **L** and **N** are presented as the mean ± SEM. *P < 0.05, **P < 0.01, ***P < 0.001 by 2-tailed unpaired t test.

The AOM/DSS model is an inflammation-associated CRC model, where inflammation is a potential contributor to carcinogenesis in the colon [31, 32]. To examine the role of Rap2B in the development of colonic inflammation, we injected *Rap2B*^IEC-WT^ mice and *Rap2B*^IEC-KO^ mice with AOM, followed by administration of 2.5% DSS in their drinking water for 3 days to establish an acute colitis model (Fig. 3A). As expected, *Rap2B*^IEC-KO^ mice exhibited less weight loss (Fig. 3B), reduced rectal bleeding (Fig. 3C) and less diarrhea (Fig. 3D) compared to *Rap2B*^IEC-WT^ mice, along with improved histological outcomes (Fig. 3E, F). Moreover, the colons of *Rap2B*^IEC-KO^ mice displayed significantly decreased expression of Ki67 (Fig. 3G, H), the macrophage marker F4/80 (Fig. 3I, J) and the neutrophil marker S100A9 (Fig. 3K, L) compared to those of WT mice. These data demonstrate that Rap2B deficiency reduces susceptibility to colonic inflammation.

### Rap2B interacts with plectin

**Fig. 4:**
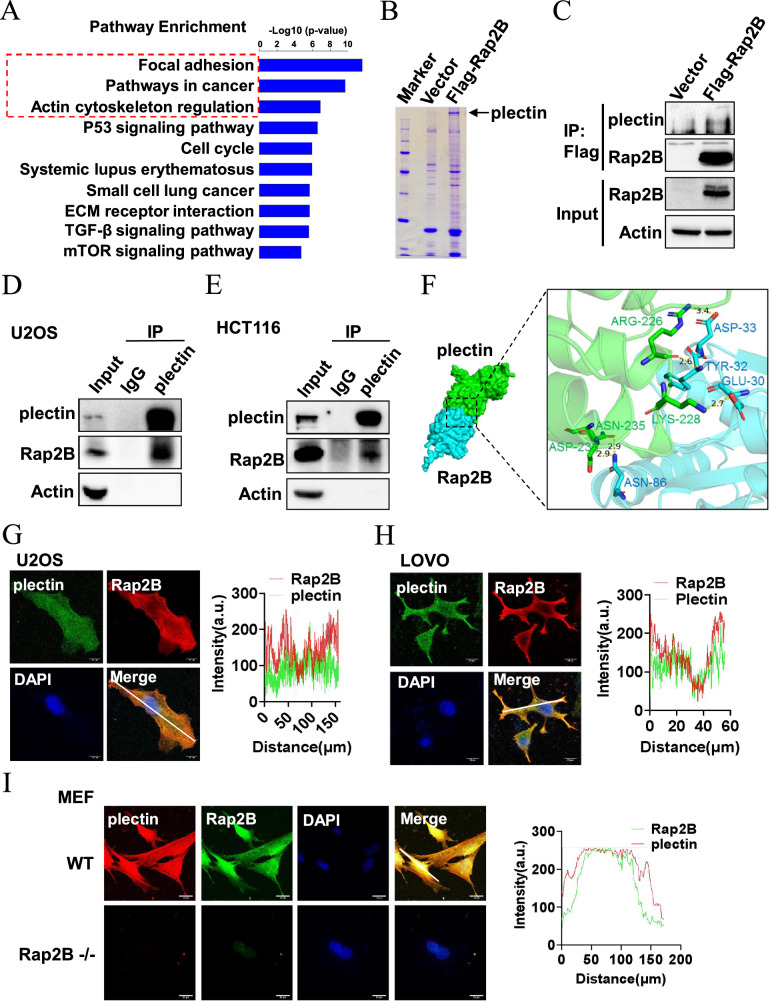
Rap2B interacts with plectin. **A** The most prominent KEGG pathways identified from the comparison between the control group and the Rap2B overexpression group. **B** Mass spectrometry analysis of Rap2B binding proteins. Extracts from U2OS cells infected with the indicated adenovirus for 2 days were immunoprecipitated with Flag-beads, resolved by SDS-PAGE, and visualized by Coomassie blue staining. Plectin was identified based on peptide sequences obtained from the mass spectrometry. **C** Coimmunoprecipitation (co-IP) of ectopically expressed Rap2B with endogenous plectin in HCT116 cells. **D**, **E** Co-IP of endogenous Rap2B and plectin in U2OS and HCT116 cells. **F** Molecular docking predictions indicate a possible interaction of between Rap2B and the ABD domain of plectin protein. The ligand binding site is visualized in 3D using PyMOL, with interacting residues labeled and specific H-bonds presented in ruby. **G**, **H** Endogenous Rap2B co-localizes with plectin in U2OS and HCT116 cells. **I** Endogenous plectin co-localizes with Rap2B in WT but not in Rap2B-/- MEF cells.

To gain a comprehensive understanding of the signaling pathways affected by Rap2B, we employed RNAseq to investigate differentially expressed genes between the vector control and Rap2B overexpression group. Kyoto Encyclopedia of Genes and Genomes (KEGG) pathway enrichment analysis revealed significant enrichment in biological pathways related to focal adhesion, cancer pathways and actin cytoskeleton regulation (Fig. 4A). To explore how Rap2B regulates these biological pathways, we conducted immunoprecipitation coupled to mass spectrometry (IP-MS) to identify its binding partners. Notably, mass spectrometry identified the cytoskeletal linker plectin as a key interacting partner of ectopic Flag-tagged Rap2B in U2OS cells (Fig. 4B). Subsequent validation in HCT116 cells confirmed a strong interaction between Rap2B and plectin (Fig. 4C), which was further corroborated by the co-immunoprecipitation between endogenous Rap2B and plectin in U2OS osteosarcoma cells and LOVO colorectal cancer cells (Fig. 4D, E). We next predicted the potential interaction sites between Rap2B and plectin using molecular docking. The actin-binding domain (ABD) of plectin, specifically residues Asn-233, ASN-235, LYS-228, and ARG-226 formed hydrogen bonds with residues ASN-86, GLU-30, TYR-32, and ASP-33 of Rap2B (Fig. 4F). To further assess the association between Rap2B and plectin, we performed immunofluorescence assays to examine their subcellular localization. Notably, Rap2B co-localized with plectin in the cytoplasm and cell membrane (Fig. 4G, H). Interestingly, while Rap2B also co-localized with plectin in wild-type (WT) mouse embryonic fibroblasts (MEFs), plectin expression diminished along with Rap2B deletion in Rap2B knockout MEFs (Fig. 4I), suggesting that Rap2B may influence plectin expression through their interaction.

### Rap2B upregulates plectin through its CAAX motif

**Fig. 5:**
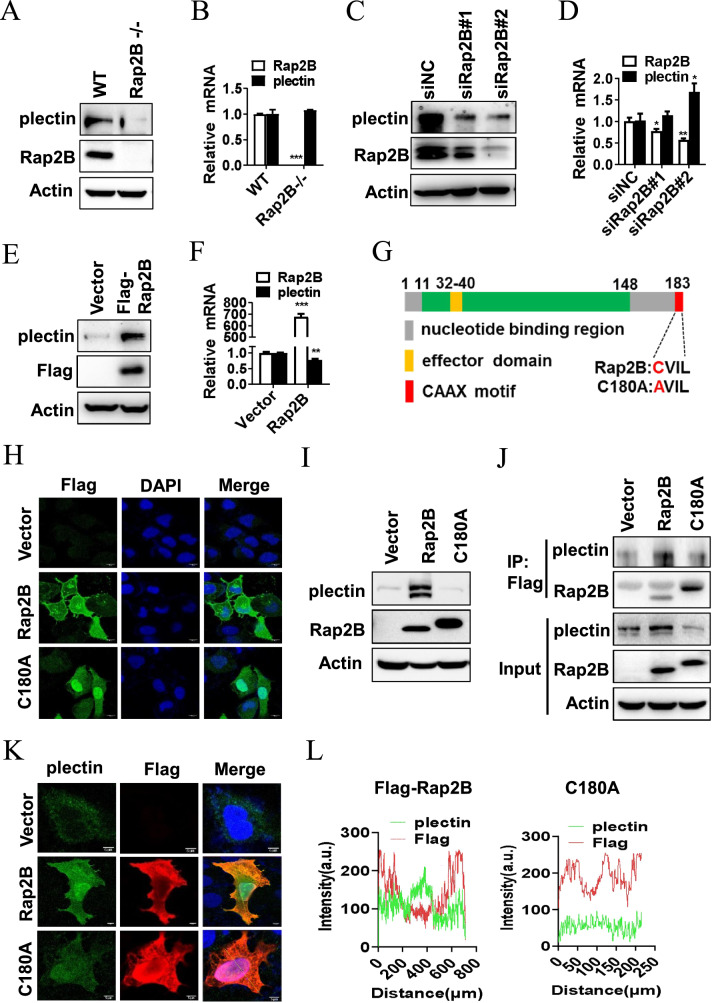
Rap2B upregulates plectin through CAAX motif. **A** The protein levels of plectin, Rap2B, and actin were measured by Western blot in WT and Rap2B-/- MEF cells. **B** The mRNA levels of plectin and Rap2B were measured by qRT-PCR in WT and Rap2B-/- MEF cells. **C** The levels of plectin, Rap2B, and actin were measured by Western blot in LOVO cells transfected with Rap2B siRNA. **D** The mRNA levels of plectin and Rap2B were measured by qRT-PCR in LOVO cells transfected with Rap2B siRNA. **E** The levels of plectin, Rap2B, and actin were measured by Western blot in LOVO cells infected with Rap2B adenovirus. **F** The mRNA levels of plectin and Rap2B were measured by qRT-PCR in LOVO cells infected with Rap2B adenovirus. **G** Domain structure of Rap2B and the scheme of the C180A mutant. **H** The localization of Rap2B and the C180A mutant were detected by immunofluorescence staining. **I** The levels of plectin, Rap2B, and actin were measured by Western blot in LOVO cells infected with Rap2B WT or C180A adenovirus. **J** Co-IP of ectopically expressed Rap2B and the C180A mutant with endogenous plectin in LOVO cells. **K**, **L** The co-localization of Rap2B and the C180A mutant with plectin in LOVO cells. Data in **B**, **C**, **E**, **F**, **H**, **I** are presented as the mean ± SEM. *P < 0.05, **P < 0.01, ***P < 0.001 by 2-tailed unpaired t test.

We then evaluated plectin expression in the presence or absence of Rap2B. Consistent with the above immunofluorescence results, plectin protein levels were substantially reduced in Rap2B-deficient MEFs or in LOVO cells with Rap2B knockdown (Fig. 5A, C). However, mRNA levels of plectin remained unchanged with Rap2B manipulations (Fig. 5B, D). In contrast, overexpression of Rap2B resulted in a significant increase in plectin protein levels but not its mRNA (Fig. 5E, F), suggesting that Rap2B may modulate plectin expression at the post-transcriptional level.

The C-terminal CAAX motif anchors Rap2 to cell membranes [33]. To determine whether the membrane localization of Rap2B is essential for its function in regulating plectin, we constructed a Rap2B C180A mutant to disrupt its plasma membrane localization, and cells expressing the C180A mutant exhibited prominent nuclear staining (Fig. 5G, H). To verify the importance of the CAAX motif in plectin upregulation, we examined plectin expression following the overexpression of both WT Rap2B and the C180A mutant. The results indicated that overexpression of Rap2B significantly increased plectin protein levels, while the C180A mutant did not (Fig. 5I). Furthermore, the Rap2B C180A mutant disrupted the interaction and co-localization between plectin and Rap2B (Fig. 5J–L). Overall, these findings suggest that Rap2B upregulates plectin protein expression through its CAAX motif.

### Rap2B inhibits the polymerization of F-actin through plectin

**Fig. 6:**
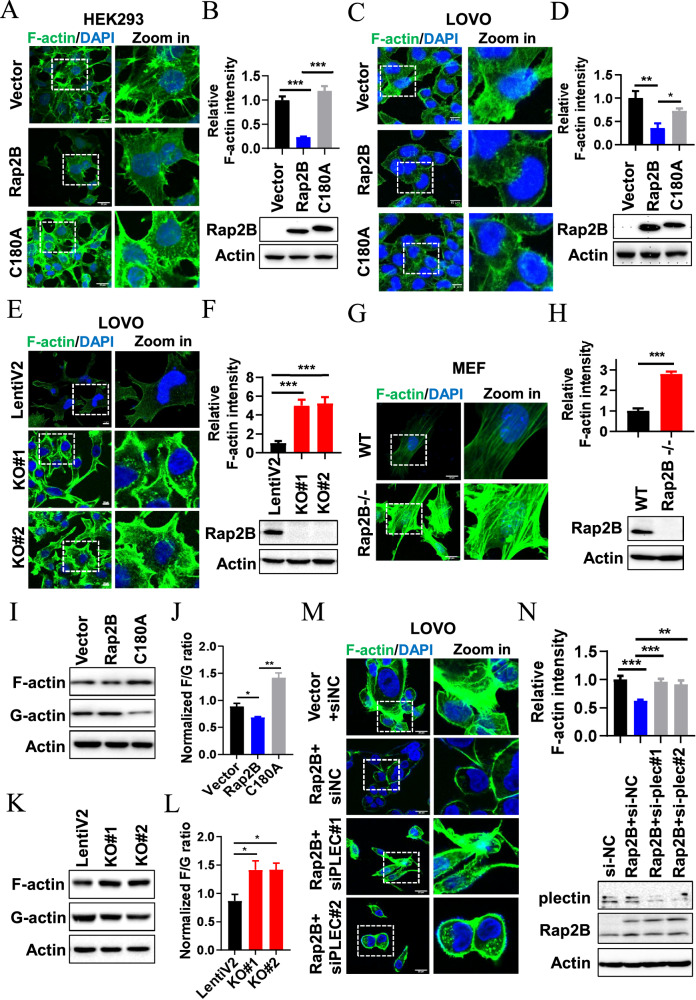
Rap2B inhibits the polymerization of F-actin through plectin. **A** Overexpression of Rap2B, but not the C180A mutant, inhibits F-actin assembly in HEK293 cells. **B** Relative F-actin intensity were quantified, and Rap2B expression were analyzed by WB in HEK293 cells infected with vector, Flag-Rap2B and Flag-Rap2B C180A mutant adenovirus. **C** Overexpression of Rap2B, but not the C180A mutant, inhibits F-actin assembly in LOVO cells. **D** Relative F-actin intensity were quantified, and Rap2B expression were analyzed by WB in LOVO cells infected with vector, Flag-Rap2B and Flag-Rap2B C180A mutant adenovirus. **E** Rap2B knockout stimulates F-actin assembly in LOVO cells. **F** Relative F-actin intensity were quantified, and Rap2B expression were analyzed by WB in Rap2B stable knockout LOVO cells. **G** Rap2B knockout stimulates F-actin assembly in MEF cells. **H** Relative F-actin intensity were quantified, and Rap2B expression were analyzed by WB in WT and Rap2B knockout MEF cells. **I**, **J** Effect of Rap2B and C180A overexpression on actin polymerization was examined using the G-actin/F-actin In Vivo Assay Kit. **K**, **L** Effect of Rap2B knockout on actin polymerization was examined using the G-actin/F-actin In Vivo Assay Kit. **M** Knockdown plectin restored F-actin signal reduced by Rap2B overexpression. **N** LOVO cells were transfected with si-NC or si-PLEC for 24 h, and then the cells were infected with Rap2B or control adenovirus for another 24 h, respectively. The cells were fixed and stained for F-actin. Relative F-actin intensity were quantified, and Rap2B expression were analyzed by WB. Data in **B**, **D**, **F**, **H**, **J**, **L** are presented as the mean ± SEM. *P < 0.05, **P < 0.01, ***P < 0.001 by 2-tailed unpaired t test.

In addition to its primary role in scaffolding and mechanical stabilization, plectin is a crucial regulator of cellular processes involving actin cytoskeleton dynamics [29]. The absence of plectin in cultured mouse fibroblasts leads to an increase in actin stress fibers [29]. To evaluate the effect of Rap2B on actin dynamics, we expressed WT Rap2B and the C180A mutant in HEK293 and LOVO cells, followed by phalloidin staining to visualize F-actin. Notably, phalloidin staining revealed a significant decrease in F-actin fluorescence in WT Rap2B-expressing cells compared to control cells, while the collapse of F-actin caused by Rap2B was partially restored in cells expressing the C180A mutant (Fig. 6A–D). Furthermore, knockout of Rap2B in LOVO cells resulted in a significant increase in phalloidin staining (Fig. 6E, F). Consistently, Rap2B knockout in MEF cells also showed a marked increase in F-actin levels compared to WT MEFs (Fig. 6G, H). We then analyzed the F/G-actin ratios using Western blotting. Overexpression of Rap2B, but not the C180A mutant, significantly decreased the F/G-actin ratios (Fig. 6I, J), while Rap2B knockout cells exhibited increased F/G-actin ratios (Fig. 6K, L). More importantly, knockdown of plectin significantly restored the F-actin signal reduced by Rap2B overexpression (Fig. 6M, N). Collectively, these data indicate that Rap2B inhibits F-actin polymerization through upregulating plectin expression.

### Rap2B promotes colorectal cancer cell proliferation and migration depending on plectin-F-actin axis

**Fig. 7:**
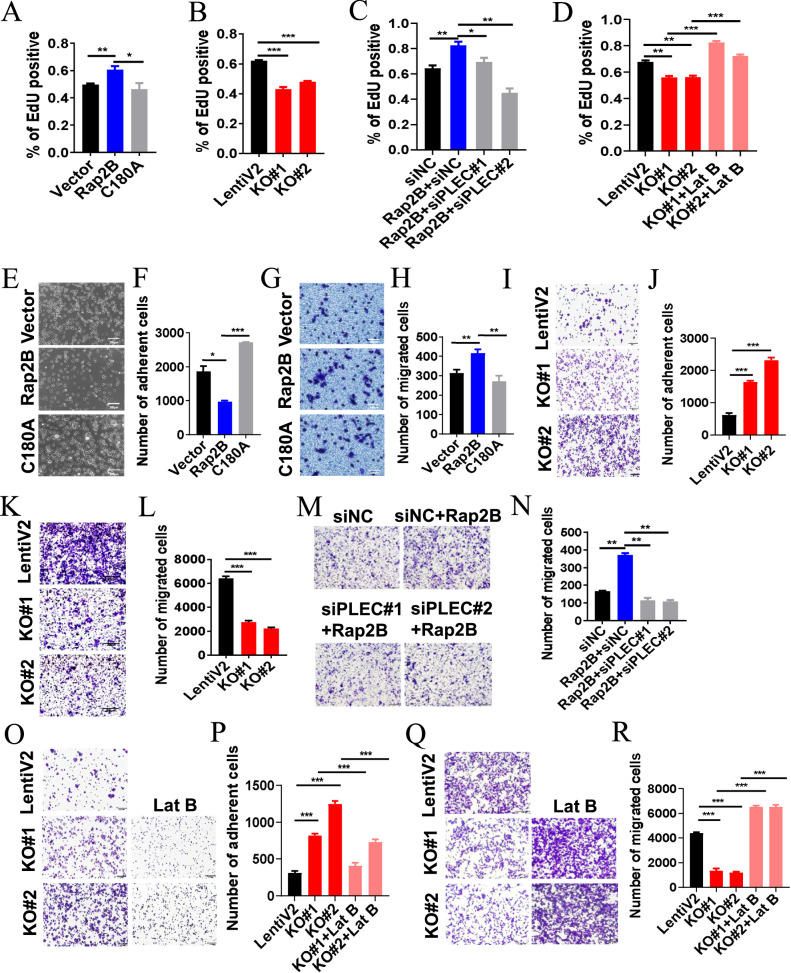
Rap2B promotes colorectal cancer cell proliferation and migration depending on plectin-F-actin axis. **A** The effects of Rap2B and C180A overexpression on the proliferation of LOVO cells was examined by EdU assay. **B** The effects of Rap2B knockout on the proliferation of LOVO cells was examined by EdU assay. **C** LOVO cells were transfected with si-NC or si-PLEC for 24 h, and then the cells were infected with Rap2B or control adenovirus for another 24 h, respectively. The proliferation of these cells was evaluated using the EdU assay. **D** LOVO cells with Rap2B knockout were treated with Lat B or not, and then the proliferation of these cells was assessed using the EdU assay. **E**, **F** The effects of Rap2B and C180A overexpression on the ability of LOVO cell adhesion was examined through a cell adhesion assay. **G**, **H** The effects of Rap2B and C180A overexpression on the ability of LOVO cell migration was examined using a transwell assay. **I**, **J** The effects of Rap2B knockout on the ability of LOVO cell adhesion was examined using a cell adhesion assay. **K**, **L** The effects of Rap2B knockout on the ability of LOVO cell migration was examined using a transwell assay. **M**, **N** LOVO cells were transfected with si-NC or si-PLEC for 24 h, and then the cells were infected with Rap2B or control adenovirus for another 24 h, respectively. The cell adhesion abilities were examined using a cell adhesion assay. **O**–**R** LOVO cells with Rap2B knockout were treated with Lat B or not, and then the cell adhesion and migration abilities of these cells were examined using a cell adhesion assay and transwell assay.

To further characterize the role of Rap2B in CRC, we examined its impact on the biological behaviors of CRC cell lines. Results from the EdU assay indicated that overexpression of Rap2B, but not the C180A mutant, promoted the proliferation of LOVO cells (Fig. 7A, Fig. S2A). In contrast, knockdown of Rap2B significantly suppressed the proliferation of these cells (Fig. 7B, Fig. S2B). Importantly, the accelerated cell proliferation in Rap2B-overexpression cells was compromised by plectin knockdown (Fig. 7C, Fig. S2C). Moreover, the retarded cell proliferation in Rap2B-deficient LOVO cells was restored upon treatment with Latrunculin B (Lat B), which induces depolymerization of F-actin [34] (Fig. 7D, Fig. S2D). These findings suggest that plectin-F-actin signal is required for Rap2B-driven CRC cell proliferation.

We next investigated whether Rap2B affects CRC cell motility. Unlike the C180A mutant, Rap2B overexpression significantly inhibited cell adhesion and promoted cell migration compared to control cells (Fig. 7E–H). In contrast, Rap2B deletion promoted cell adhesion and inhibited cell migration compared with control cells (Fig. 7I–L). Importantly, knockdown plectin reversed the increased cell migration-driven by Rap2B overexpression (Fig. 7M, N). Additionally, Lat B treatment restored cell adhesion and migration ability which were attenuated by Rap2B knockout (Fig. 7O–R). These data further indicate that plectin-F-actin axis is also essential for the elevated metastatic potential in Rap2B-high CRC cells in vitro.

### Plectin positively correlates with Rap2B and predicts malignant progression of CRC

**Fig. 8:**
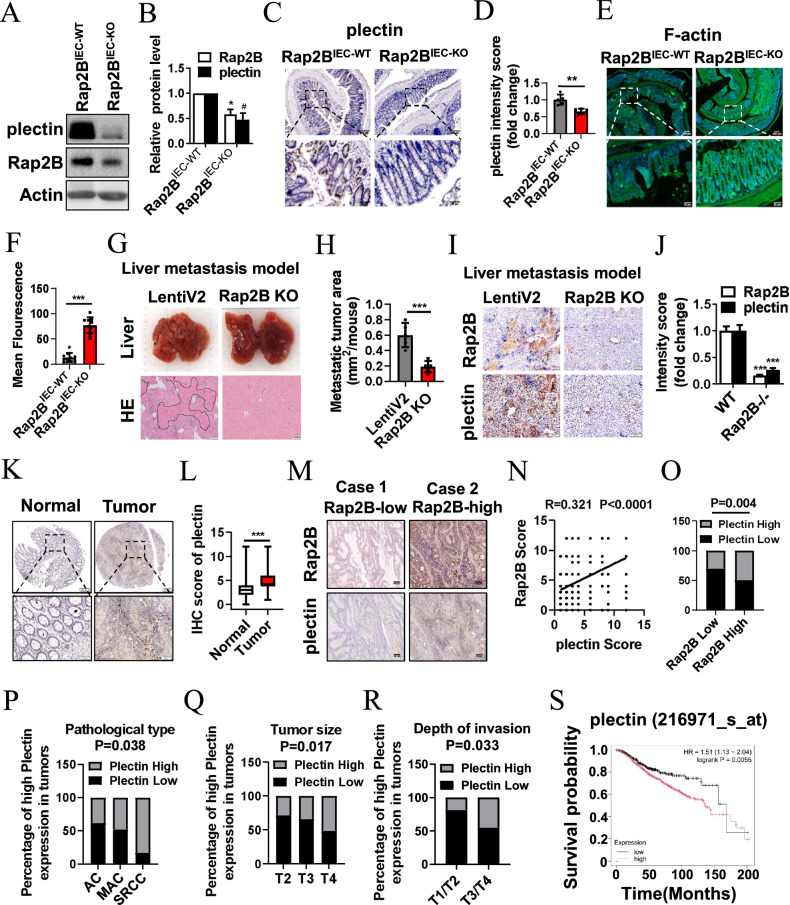
Plectin positively correlates with Rap2B and predicts malignant progression of CRC. **A**, **B** Immunoblots analysis of Rap2B and plectin using lysates from colon epithelial cells of *Rap2B*^IEC-WT^ and *Rap2B*^IEC-KO^ mice in the CRC induction model. **C**, **D** IHC staining for plectin in colons from *Rap2B*^IEC-WT^ and *Rap2B*^IEC-KO^ mice with CRC induction model (n = 5). **E**, **F** Phalloidin staining for F-actin in colons from *Rap2B*^IEC-WT^ and *Rap2B*^IEC-KO^ mice with CRC induction model (n = 5; 2 images/mice). **G** Representative images of mouse liver tissue with metastatic tumors and H&E staining on liver tissue sections. The black dashed lines indicate the tumor borders. **H** Quantification of metastatic tumor areas (n = 5). **I**, **J** Representative images and quantifications of IHC staining for plectin and Rap2B in liver metastasis sections (n = 5). **K**, **L** Representative images and IHC intensity analysis using plectin antibody in 179 human colon cancer and 161 adjacent normal colon tissue. **M** Representative images of Rap2B and plectin expressions in Rap2B-low case and Rap2B-high case are presented. **N** Correlation between plectin and Rap2B expression were examined using Pearson correlation coefficient test (n = 360). **O** Correlation between plectin and Rap2B expression were examined by Fisher’s exact test. Percentages of high level of plectin expression correlated with pathological type (**P**), tumor sizes (**Q**), and depth of invasion (**R**) were examined by χ2 test. **S** High plectin expression correlated with overall survival for 979 patients with CRC (High plectin patients 617, Low plectin patients 362) (P = 0.0055, log-rank test). Data are presented as mean ± SEM. *P < 0.05, **P < 0.01, ***P < 0.001 by 2-tailed unpaired t test.

To further determine whether plectin is involved in the regulation of IEB and CRC progression conducted by Rap2B in vivo, we analyzed the expression of the two genes at the tissue level. As shown in Fig. 8A–D, the protein expression levels of plectin were significantly decreased in the colon of *Rap2B*^IEC-KO^ mice compared to controls. Consistent with our in vitro results, F-actin levels in the colon were significantly increased in the AOM/DSS-induced CRC model of *Rap2B*^IEC-KO^ mice compared to controls (Fig. 8E, F). Liver metastasis is commonly observed in CRC patients, contributing to high mortality rates. To explore the role of Rap2B in CRC metastasis, we established a CRC liver metastasis model by tail vein injection of LOVO cells with or without Rap2B knockout. Rap2B knockout led to reduced liver metastasis (Fig. 8G, H), and IHC staining of metastatic liver tissue revealed decreased plectin expression following Rap2B knockout (Fig. 8I, J). These results indicate that Rap2B and plectin may serve as prognostic markers for CRC metastasis, and the plectin-F-actin pathway plays an important role in Rap2B-associated CRC progression.

To assess the clinical significance of our findings, we conducted IHC analyses on TMA slides of CRC using a plectin antibody. Our results showed that plectin was expressed at significantly higher levels in CRC tissues compared to adjacent normal colon tissues (Fig. 8K, L). Additionally, we observed a significant positive correlation between the expression of plectin and Rap2B in CRC patients (Fig. 8M, N), with elevated levels of Rap2B expression positively correlating with increased plectin levels (Fig. 8O). Next, we analyzed the relationship between plectin levels and various clinicopathological characteristics in human CRC specimens. Our findings revealed that elevated plectin levels were positively associated with specific pathological types (Fig. 8P), tumor sizes (Fig. 8Q), and the depth of invasion (Fig. 8R), as summarized in Table 2. Among the different histological types of CRC, adenocarcinoma (AC) accounts for over 90% of cases and is associated with a better prognosis compared to mucinous adenocarcinoma (MAC) and signet ring cell colorectal cancer (SRCC), both of which are rare CRC subtypes that correlate with more advanced tumor stages and increased metastatic potential [35]. Notably, increased plectin expression was observed in 38.7% of patients diagnosed with AC, while this percentage increased to 48% in patients with MAC and exceeded 80% in those with SRCC, suggesting that plectin expression may serve as a prognostic indicator of CRC malignancy. Additionally, data from the Kaplan Meier plotter indicated that high plectin expression is associated with poor overall survival in CRC patients (Fig. 8S). These results strongly support the involvement of Rap2B and plectin in the clinical behavior of CRC patients, supporting our hypothesis that Rap2B promotes tumorigenesis and the progression of CRC through plectin in vivo.

**Table 2:** Plectin staining and clinicopathological characteristics of 179 CRC patients.

	Plectin staining				
Variables	Low (%)	High (%)		Total	*P* value
**Gender**					
Male	55 (59.78)	37 (40.22)		92	0.288
Female	48 (54.55)	40 (45.45)		88	
**Age**					
<57	29 (63.04)	17 (36.96)		46	0.227
≥57	74 (55.22)	60 (44.78)		134	
**Tumor size**					
T2	10 (71.43)	4 (28.57)		14	0.017
T3	49 (65.33)	26 (34.67)		75	
T4	44 (48.35)	47 (51.65)		91	
**Lymph node metastasis**					
N0	65 (60.19)	43 (39.81)		108	0.345
N1	24 (53.33)	21 (46.67)		45	
N2	14 (51.85)	13 (48.15)		27	
**Distant metastasis**					
Negative	99 (57.56)	73 (42.44)		172	0.471
Positive	4 (50.00)	4 (50.00)		8	
**Depth of invasion**					
T1/T2	13 (81.25)	3 (18.75)		16	0.033
T3/T4	90 (54.88)	74 (45.12)		164	
**Clinical staging**					
1	8 (66.67)	4	(33.33)	12	0.176
2	56 (60.22)	37 (39.78)		93	
3	35 (53.03)	31 (46.97)		66	
4	4 (44.44)	5 (55.56)		9	
**Clinical grading**					
1	1 (100.00)	0 (0.00)		1	0.038
1–2	0 (0.00)	5 (100.00)		5	
2	71 (60.17)	47 (39.83)		118	
2–3	24 (54.55)	20 (45.45)		44	
3	7 (58.33)	5 (41.67)		12	
**Pathologic typing**					
Adenocarcinoma	76 (61.29)	48 (38.71)		124	0.038
Mucinous adenocarcinoma	26 (52.00)	24 (48.00)		50	
Signet ring cell carcinoma	1 (16.67)	5 (83.33)		6	

## Discussion

In this study, we identified Rap2B as a critical regulator of CRC tumorigenesis and progression using intestinal epithelial-specific knockout mice. Mechanistically, Rap2B binds plectin via its CAAX motif, upregulating plectin expression and suppressing F-actin assembly, which is essential for CRC cell proliferation and metastasis. Importantly, elevated Rap2B and plectin expression correlate with poor CRC patient prognosis. Collectively, these findings elucidate a novel Rap2B-plectin-F-actin axis driving CRC tumorigenesis and progression, refining mechanistic insights into Ras-family-mediated cytoskeletal remodeling in cancer pathogenesis.

Dysfunction and deregulation of the RAS family of small GTPases play crucial roles in cell proliferation and apoptosis, migration, cytoskeletal remodeling and vesicle trafficking [36]. The Rap subfamily, which includes Rap1 and Rap2, shares a high degree of sequence and structure homology with Ras proteins [37]. The isoform Rap2B was initially cloned from a platelet cDNA library [16], and is located at 3q25.2 on the human chromosome, a known hotspot in cancer research. Rap2B is a highly conserved protein, with identical amino acid sequences found in several species, including Homo sapiens, Castor canadensis, Macaca mulatta, Mus musculus, Pan troglodytes, Pteropus alecto and Rattus norvegicus (Fig. S3). Although Rap2B exhibits upregulated expression in various cancer types, it displays a notably low incidence of somatic mutations in tumors-a striking contrast to the high mutational frequency observed in RAS proteins-indicating that the maintenance of high level of wild type Rap2B is somehow important for cancer development. Although accumulating evidence from our studies and others suggests that Rap2B contributes to tumor progression by modulating critical cellular processes, including proliferation, migration, invasion, and survival, the precise molecular mechanisms driving its oncogenic activity remain incompletely understood [17, 20, 23, 26]. While Rap2B has been implicated in key signaling cascades such as MAPK, PI3K/AKT, and cytoskeletal reorganization pathways, its context-dependent regulatory dynamics and mechanistic contributions to these networks are not yet fully characterized. Furthermore, the specific functions and related mechanisms of Rap2B in vivo remain unexplored.

The Guan group reported that Rap2 functions as a molecular switch in mechanotransduction, with the deletion of Rap2 blocking the regulation of YAP and TAZ by stiffness signals [12]. Our RNAseq data also indicated that Rap2B was mainly involved in the regulation of focal adhesion, actin cytoskeleton regulation and cancer pathways, suggesting that Rap2B may play a pivotal role in mechanical signal transduction, particularly in cytoskeletal remodeling and cell adhesion. The integration of cytoskeletal and adhesive networks is critical for maintaining the integrity of the epithelial monolayer. Disruption of the actin cytoskeleton can compromise the intestinal epithelial barrier, potentially leading to colitis and increasing the risk of colorectal cancer. Consequently, we constructed intestinal epithelial-specific knockout mice for Rap2B to investigate its role in tumorigenesis and actin cytoskeleton regulation, using CRC as a model in this study.

The TCGA database and our IHC analysis of TMA revealed that Rap2B is highly expressed in CRC, with elevated Rap2B protein levels correlating with poor overall survival in CRC patients. However, the specific mechanism by which Rap2B functions in CRC remains to be elucidated. CRC is the third most common cancer globally [38]. Despite advancements in treatment, both the incidence and mortality rates of CRC continue to rise [39], highlighting the urgent need for the discovery of additional molecular biomarkers of CRC for early detection and targeted therapy. In our study, we employed a DSS/AOM colitis and CRC induction model, and innovatively found that IEC-specific Rap2B deletion inhibited colitis progression and CRC tumorigenesis in mice. Mechanistically, we observed that knockout of Rap2B led to an increase in F-actin assembly. F-actin is a critical component for maintaining the integrity and function of the gastrointestinal mucosal barrier [6, 40]. Our findings suggest that Rap2B inhibits the polymerization of F-actin, thereby affecting intestinal barrier integrity and tumor cell migration, ultimately promoting the development and progression of CRC. Notably, previous studies in diverse cancer models have identified Rap2B as a multifunctional GTPase that directly drives tumorigenic processes, such as enhancing proliferation via activation of the ERK/MAPK and PI3K/Akt signaling pathways [21–23, 41]. Beyond its established role in inflammation-driven tumorigenesis discussed in this context, Rap2B’s intrinsic oncogenic functions may synergize with its involvement in intestinal epithelial cell IEC injury, thereby exacerbating CRC progression.

To gain mechanistic insight into the role of Rap2B in actin cytoskeleton regulation, we identified plectin as a direct interactor of Rap2B utilizing immunoprecipitation followed by mass spectrometry. Plectin, a cytolinker protein belonging to the plakin family, mediate physical linkages among diverse cytoskeletal systems, including microtubules (MTs), actin microfilaments, and intermediate filaments (IFs) [42, 43]. This 500 kDa protein features contained an N-terminal canonical actin-binding domain (ABD), a central rod domain and a C-terminal IF-binding domain (IFBD) [27]. As a major linker and scaffolding protein of the cytoskeleton, plectin deficiency results in a substantial increase in the number of actin stress fibers and focal adhesion contacts, with minimal or no changes in the organization of the MT and IF networks [29].

In recent years, plectin has emerged as a potential oncogenic driver in multiple human cancers [27, 28, 44]. Aberrant plectin expression has often been found across multiple cancer types [28]. As a pivotal structural protein and cytoskeletal cross-linker, plectin regulates broad cancer cell behaviors, including growth, adhesion, migration, invasion, apoptosis, EMT, pleomorphism, micronucleus formation, and centrosome localization, all crucial for tumor initiation and progression [45–50]. Notably, its mis-localization to the cell surface in malignancies has positioned plectin as a diagnostic biomarker and prognostic indicator [28, 51, 52]. Despite these advances, the specific molecular mechanisms underlying its role in distinct stages of various cancers remain poorly elucidated.

In this study, we report that Rap2B interacts with plectin and upregulates plectin expression. Recent findings suggest that the primary contribution of RAS family to protein expression might occur at the level of mRNA translation rather than gene transcription [53, 54]. Our results also indicate that Rap2B promotes plectin expression through its CAAX motif. However, the precise mechanisms by which Rap2B upregulates plectin expression require further investigation. Moreover, we found that Rap2B inhibits the polymerization of F-actin through upregulating plectin. Finally, we demonstrated that the regulatory effects of Rap2B on CRC are associated with the plectin-F-actin pathway, both in vitro and in vivo.

Taken together, we firstly reported the essential role of Rap2B for the initiation and progression of CRC using IEC-specific Rap2B knockout mice. Additionally, we have highlighted Rap2B’s involvement in cytoskeletal regulation. We believe that our finding will enhance the understanding of the regulatory mechanisms of the Ras family on actin cytoskeleton, and provide a new target for the diagnosis and treatment of CRC.

## Materials and methods

### Patients and tissue samples

A tissue microarray (TMA) comprising 179 cases of CRC tissues and 161 adjacent noncancerous tissues was purchased from Shanghai Xinchao Biotechnology (Shanghai, China). Among these, 161 tumor tissues and paired normal tissues are collected from same patients. Clinicopathological data, including age, gender, tumor size, lymph node metastasis, distant metastasis and depth of invasion were obtained from patients’ medical records. The TMA was constructed with a core diameter of 1.5 mm, where each core corresponded to a distinct tissue specimen meticulously selected and histologically validated by pathologists. Ethical approval for this study was approved by the Ethics Committee of Shanghai Xinchao Biotechnology Co., Ltd, and informed consent was obtained from all individuals.

### Mice and genotyping

*Rap2B* ^flox/flox^ mice with loxP sites flanking entire Rap2B exon in C57BL/6 genetic background were intercrossed with *Villin*-Cre mice to obtain *Rap2B*^WT^; *Villin*-Cre (*Rap2B*^IEC-WT^) and *Rap2B* ^flox/flox^; *Villin*-Cre (*Rap2B*^IEC-KO^) mice. All mouse lines were maintained on a C57Bl/6 genetic background. Toe tissues of one-week old mice were cut for genotyping. 2 μL of purified genomic DNA was used in the genotyping PCR reaction, and agarose gel electrophoresis was used to detect the amplified products. All animal experimental procedures were approved by the Animal Ethical and Welfare Committee of Xuzhou Medical University.

### AOM/DSS model

*Rap2B*^IEC-WT^ and *Rap2B*^IEC-KO^ mice were confirmed by genotyping. For tumor induction, 8-week-old male mice were injected once with 10 mg/kg AOM (Sigma) intraperitoneally (i.p.) on the day 0 of the experiment. On day 7, 2% DSS (MP Biologicals) was given in drinking water for 1 week followed by regular drinking water for 2 weeks. This cycle of 1 week of DSS followed by 2 weeks of water was repeated twice. Mice were sacrificed at 70 days after AOM injection. For colitis induction, 8-week-old mice were similarly injected once with 10 mg/kg AOM i.p. on the day 0, and they were sacrificed after receiving 2% DSS in their drinking water for 3 days.

### Tumor metastasis model

Female nude mice (Balb/c) aged 6–7 weeks were randomly distributed into two groups, and were injected to the tail veins with 100 µL indicated LOVO cell suspension (2 × 10^6^ cells/mL). The mice were sacrificed at 30 days. The livers were collected and weighed for in vivo tumor metastasis analysis. The number of metastatic liver nodules was counted and further confirmed via H&E staining.

### Cell culture and reagents

Human colorectal cell lines LOVO and HCT116, along with osteosarcoma cell line U2OS and the embryonic kidney cell line HEK-293T, were obtained from the Shanghai Cell Bank of the Chinese Academy of Sciences. These cell lines were cultured in Dulbecco’s modified Eagle’s medium (DMEM) supplemented with 10% fetal bovine serum (FBS). The cells were grown in the humidified incubator at 37 °C with 5% CO2. For cell transfection, we used jetPRIME transfection reagent according to the manufacturer’s standard protocol.

### Western blot analysis

After performing specific treatments, cells were lysed in lysed in 0.5% NP-40 buffer. Insoluble debris was pelleted by centrifugation at 13,000 × *g* for 10 min at 4 °C, and the supernatants were collected. Protein concentrations were quantified using the Brad-ford (G250) assay (KeyGEN Biotech, Nanjing, China) according to the manufacturer’s instructions. Proteins samples were separated by electrophoresis on SDS-polyacrylamide gel electrophoresis (PAGE) and then transferred to nitrocellulose filter (NC) membrane. After blocking for 1 hour in 5% milk in PBST, the membranes were incubated overnight at 4 °C with primary antibodies. Following incubation with secondary antibodies (Beyotime, Nantong, China), the immune complexes on the NC membrane were detected using an ECL kit (Advansta, CA, USA) on the Bio-Rad automatic chemiluminescence imaging system (CA, USA). The following antibodies were acquired from commercial sources: mouse anti-Rap2B (Sigma, # WH0005912M1), rabbit anti-Rap2B (GeneTex, # GTX114702) and mouse anti-plectin (Santa Cruz, #10F6). G-actin/F-actin In Vivo Assay Kit (#BK037) was purchased from Cytoskeleton.

### RNA extraction and real-time polymerase chain reaction

Total RNA was extracted from cultured cells and tissues using TRIzol reagent (15596026, Invitrogen, USA) according to the manufacturer’s instructions. Complementary DNA was synthesized using PrimeScript reverse transcription (RT) reagent kit (R323, Vazyme, China) in the presence of gDNA Eraser. Quantitative real-time polymerase chain reaction (qRT-PCR) was performed according to the instructions of SYBR Green Master Mix Kit (Q331-02, Vazyme, China), and results were calculated according to the Ct value (2^ΔΔCt^). The PCR reactions were performed using antigen-specific primers (Table S1).

### Immunoprecipitation (IP)

Indicated Cells were lysed in 0.1% ice-cold NP-40 lysis buffer, and the supernatants were transferred to a new tube. For preclearing, 1 mg of cell extracts was incubated with 50 µl of CL4B for 30 minutes. The supernatant was then incubated with appropriate primary antibodies with gentle shaking at 4 °C overnight. Following this, 12 μL of Pierce Classic Magnetic Protein A/G-beads (Thermo Scientific, CA, USA) for another 2 h at 4 °C. The beads were washed three times with cold 0.1% NP-40 lysis buffer and then resuspended in 20 μL of 1× loading buffer and boiled for 2 minutes, and analyzed by SDS-PAGE.

### Immunofluorescence staining

Cells grown on coverslips were fixed with 4% paraformaldehyde and subsequently treated with 0.1% Triton X-100 solution on ice for 5 minutes. Samples were then blocked with 3% BSA for 1 h, followed by incubation with the primary antibody at 4 °C overnight. After washing the cells three times for 5 min each with PBS, they were incubated with Alexa 488/594-conjugated secondary antibodies for 1 h. The nuclei were stained by 4’,6-diamidino-2-phenylindole (DAPI) for 5 min. Finally, the slides were mounted with 90% glycerol, and images were captured with a Zeiss Axio Observer confocal microscope.

### F-actin and G-actin ratio assay

Determination of depolymerized (free globular-G) to polymerized (filamentous-F) actin was performed using the G-actin/F-actin In Vivo Assay Kit (BK037, Cytoskeleton) according to the manufacturer’s instructions. Briefly, indicated cells were lysed in F-actin stabilization buffer and centrifuged at 350 × *g* to pellet cell debris. The supernatants were transferred to a new tube and centrifuged at 100,000 × *g* for 1 h to pellet the F-actin. After the G-actin-containing supernatant was transferred to a new tube, a depolymerization buffer was added to the pellet and incubated on ice for 1 h to depolymerize the F-actin. Following depolymerization, a 5× SDS sample buffer was added to the G-actin and F-actin-containing samples and then analyzed by Western blotting. Antibodies specific for actin were used to visualize amounts of F-actin and G-actin.

### Histopathology and IHC staining

Immediately after sacrifice, about 10 cm jejunum was removed and carefully rinsed with ice-cold saline. The tissue was either bundled with 3 M micropore tape after being cut into 6–8 sections (1 cm each) or tacked to a foam board after being opened longitudinally. Both preparations were fixed overnight in 10% formalin. Sections (5-μm) were subjected to H&E staining for histological analysis. According to the streptavidin-peroxidase method using a standard Sp Kit (PV-9001, Zhongshan biotech, Beijing, China), the standard protocol for IHC staining was done as described previously in our lab [55]. Rabbit polyclonal Rap2B antibody (GTX114702, GeneTex, 1:100), Rabbit polyclonal Ki-67 antibody (12202 s, Cell Signaling, 1:500) and mouse monoclonal plectin antibody (10F6, Santa Cruz, 1:100) were used as primary antibody and incubated overnight at 4 °C. PBS was used as a negative control in place of the primary antibody. All images were recorded by Olympus BX-51 light microscope and further processing was done using Adobe Photoshop.

### 5-ethynyl-20-deoxyuridine (EdU) assay

In total, 10,000 cells were seeded in 96-well plates and allowed to grow overnight. Following designated treatment, cells were incubated with 50 µM EdU (C10310, RiboBio, China) for 2 h. The plates were washed twice with PBS, and then treated with 0.5% TritonX-100 for 20 min after being fixed with paraformaldehyde for 30 min. Next, cells were stained with 100 µL Apollo dye solution for 20 min. The nuclei were subsequently stained with DAPI for 10 min. The proportion of cells bound to EdU was quantified by fluorescence microscopy via counting at least four random fields.

### Cell migration and adhesion assays

For the cell migration assays, cells were resuspended in 200 µL of serum-free medium and placed in the upper compartment of a transwell chamber. Migrated cells were fixed with 4% paraformaldehyde and stained with crystal violet. Five random fields were selected, and the cell number was determined under a light microscope. For the cell adhesion assays, 48-well plates were coated with 10ug/ml Fibronectin (354408, Corning, NY, USA) in minimal essential medium overnight at 4 °C. The plates were then blocked with 1% BSA for 1 h at 37 °C. After washing with PBS three times, 1 × 10^4^ LOVO cells were seeded into were seeded in each well for one hour incubation at 37 °C. After incubation, cultured cells were gently washed with PBS to remove non-adherent cells and then stained with crystal violet. Experiments were carried out in triplicate, and three random fields of each well were recorded.

### Molecular docking

The structure of the actin binding domain of human plectin was obtained from PDB (PDB ID: 1MB8), and the protein structure of human Rap2B was acquired from UniProt. PyMOL v2.3.0 was used to draw the structural models. Structural models of Rap2B with plectin were docked in silico using HDOCK. The binding site residues were identified using PyMOL v2.3.0 to demonstrate hydrophobic and hydrogen-bond interfaces.

### Statistical analysis

All data were analyzed with GraphPad Prism software (version 9.0) and are presented as the mean ± SEM. Two-way ANOVA followed by Tukey post-hoc test for multiple comparisons or two-tailed unpaired student t test were used for statistical analyses of the data when appropriate. *P < 0.05, **P < 0.01, and ***P < 0.001 were considered significant.

## Supplementary information

supplementary legends

Suppl Figure 1

Suppl Figure 2

Suppl Figure 3

Suppl Table 1

Full and uncropped western blots

## Data Availability

The published article includes all data sets generated/analyzed for this study.
