# Supplementary material for: Rap2B drives tumorigenesis and progression of colorectal cancer through intestinal cytoskeleton remodeling
Source: Cell Death Dis. 2025 Apr 13;16(1):290. doi: 10.1038/s41419-025-07627-8 (PMC11994759; doi:10.1038/s41419-025-07627-8)
Supplement: Supplementary file 1 — supplementary legends [file 41419_2025_7627_MOESM1_ESM.docx]

**Fig. S1 IEC-specific Rap2B deletion does not alter the morphological structure of colorectum.**

(**A**) Male *Rap2B*^IEC-WT^ and *Rap2B*^IEC-KO^ mice at 8 weeks of age. (**B**) Quantification of body weight of *Rap2B*^IEC-WT^ and *Rap2B*^IEC-KO^ mice (n=11). (**C** and **D**) Representative images of colons and quantification of colon length from *Rap2B*^IEC-WT^ and *Rap2B*^IEC-KO^ mice (n=11). (**E**) Representative H&E analysis of the colons from *Rap2B*^IEC-WT^ and *Rap2B*^IEC-KO^ mice. (**F** and **G**) Representative images and IHC intensity analysis for Ki-67 in tumors from *Rap2B*^IEC-WT^ and *Rap2B*^IEC-KO^ mice (n = 5).

**Fig. S2** **Rap2B promotes colorectal cancer cell proliferation depending on plectin-F-actin axis by EdU assay.**

(**A** and **B**) Representative images of EdU assay in LOVO cells with Rap2B overexpression or knockout. Red color indicates EdU-positive cells. (**C**) Representative images of EdU assay in Rap2B overexpressed LOVO cells with or without plectin knockdown. Red color indicates EdU-positive cells. (**D**) Representative images of EdU assay in Rap2B stable knockout LOVO cells with or without Lat B treatment. Red color indicates EdU-positive cells.

**Fig. S3 The Rap2B protein sequence alignment from a variety of species.**

**Table S1: List of real-time qPCR primers sets.**
