# Supplementary figures and images for: Rap2B drives tumorigenesis and progression of colorectal cancer through intestinal cytoskeleton remodeling
Source: Cell Death Dis. 2025 Apr 13;16(1):290. doi: 10.1038/s41419-025-07627-8 (PMC11994759; doi:10.1038/s41419-025-07627-8)

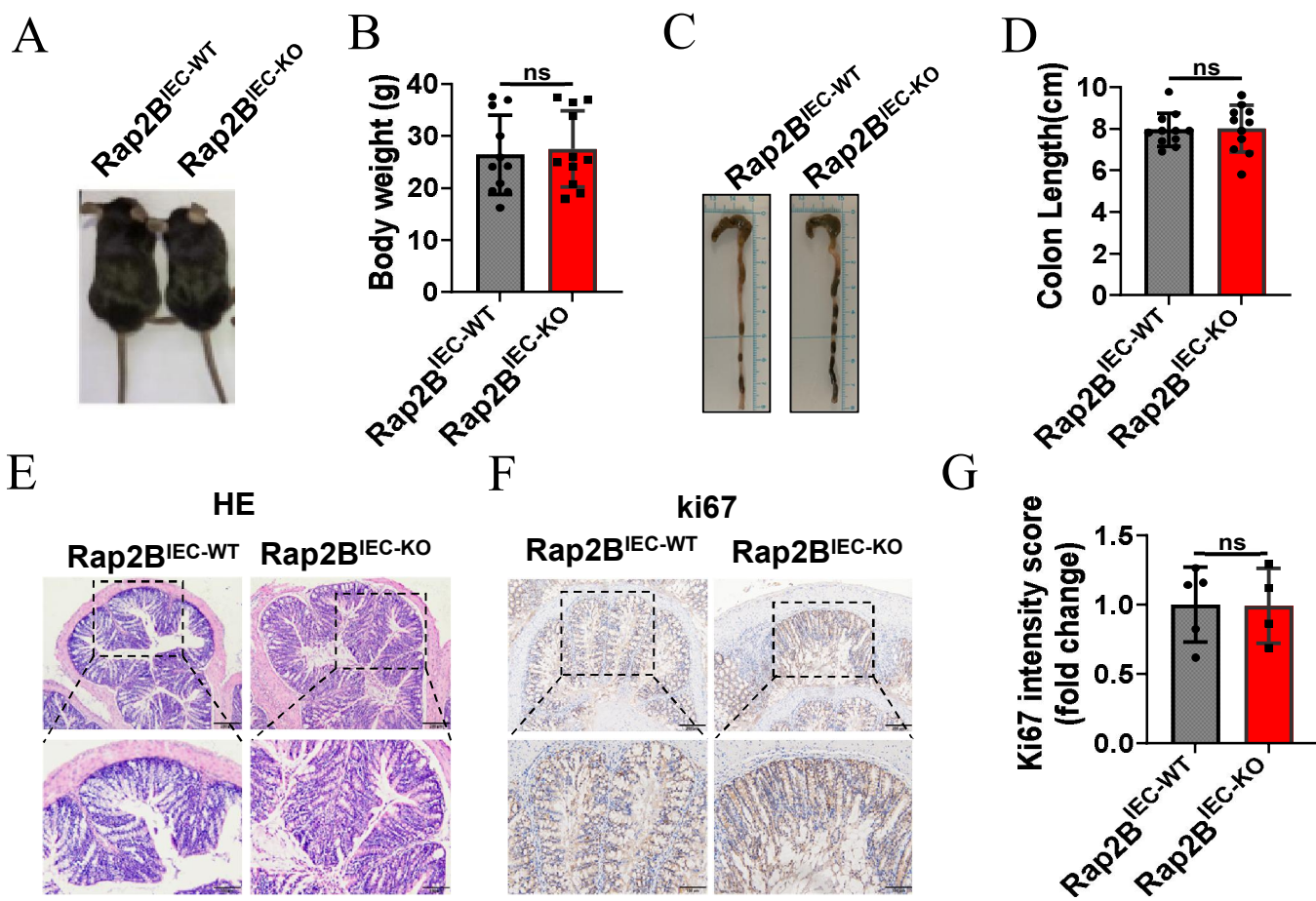

Supplement: Supplementary file 2 — Suppl Figure 1 [file 41419_2025_7627_MOESM2_ESM.pdf]

A

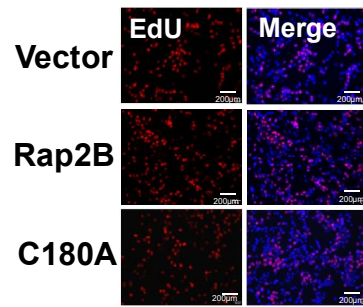

B

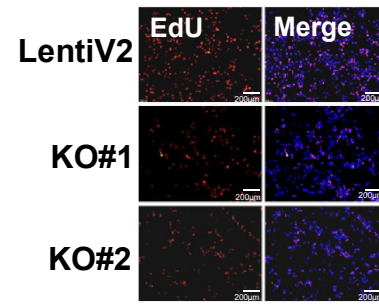

C

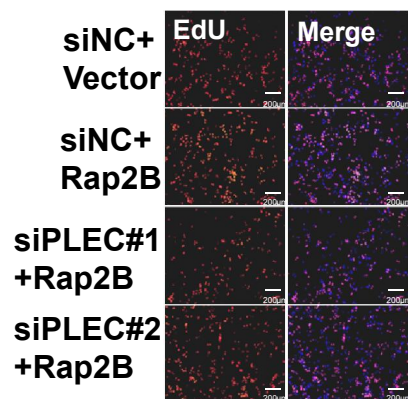

D

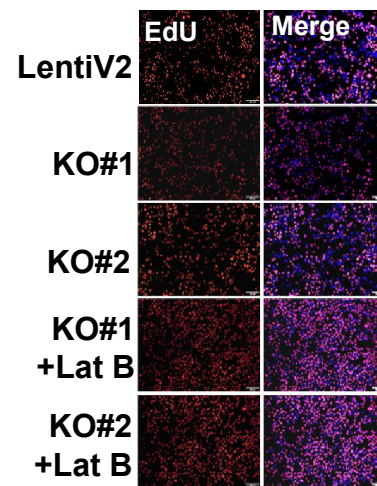

Supplement: Supplementary file 3 — Suppl Figure 2 [file 41419_2025_7627_MOESM3_ESM.pdf]
