# Supplementary material for: Rap2B drives tumorigenesis and progression of colorectal cancer through intestinal cytoskeleton remodeling
Source: Cell Death Dis. 2025 Apr 13;16(1):290. doi: 10.1038/s41419-025-07627-8 (PMC11994759; doi:10.1038/s41419-025-07627-8)
Supplement: Supplementary file 4 — Suppl Figure 3 [file 41419_2025_7627_MOESM4_ESM.pdf]

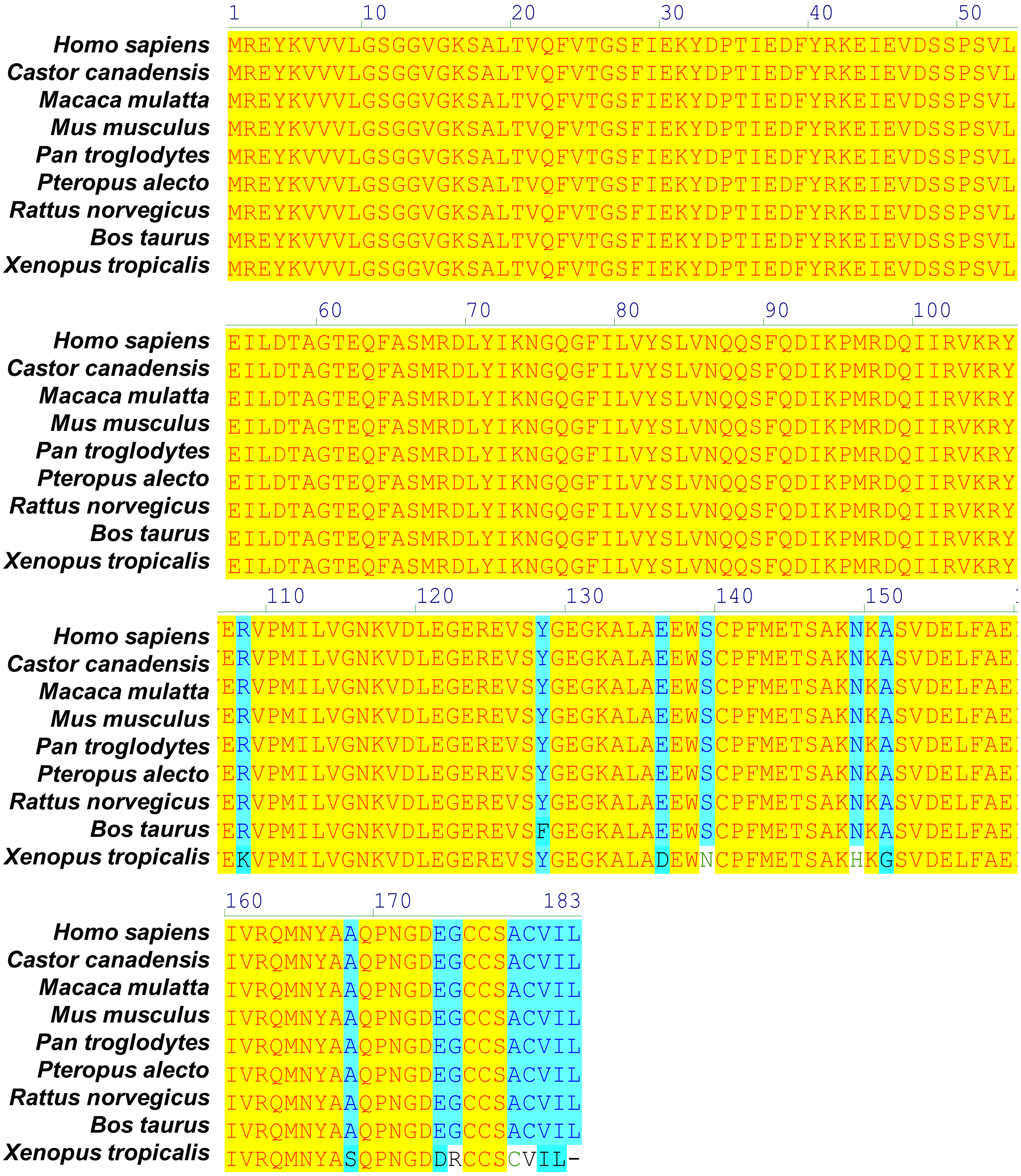
1 10 20 30 40 50
Homo sapiens MREYKVVVLGSGGVGKSALTVQFVTGSFIEKYDPTIEDFYRKEIEVDSSPSVL
Castor canadensis MREYKVVVLGSGGVGKSALTVQFVTGSFIEKYDPTIEDFYRKEIEVDSSPSVL
Macaca mulatta MREYKVVVLGSGGVGKSALTVQFVTGSFIEKYDPTIEDFYRKEIEVDSSPSVL
Mus musculus MREYKVVVLGSGGVGKSALTVQFVTGSFIEKYDPTIEDFYRKEIEVDSSPSVL
Pan troglodytes MREYKVVVLGSGGVGKSALTVQFVTGSFIEKYDPTIEDFYRKEIEVDSSPSVL
Pteropus alecto MREYKVVVLGSGGVGKSALTVQFVTGSFIEKYDPTIEDFYRKEIEVDSSPSVL
Rattus norvegicus MREYKVVVLGSGGVGKSALTVQFVTGSFIEKYDPTIEDFYRKEIEVDSSPSVL
Bos taurus MREYKVVVLGSGGVGKSALTVQFVTGSFIEKYDPTIEDFYRKEIEVDSSPSVL
Xenopus tropicalis MREYKVVVLGSGGVGKSALTVQFVTGSFIEKYDPTIEDFYRKEIEVDSSPSVL
60 70 80 90 100
Homo sapiens EILDTAGTEQFASMRDLYIKNGQGFILVYSLVNQQSFQDIKPMRDQIIRVKRY
Castor canadensis EILDTAGTEQFASMRDLYIKNGQGFILVYSLVNQQSFQDIKPMRDQIIRVKRY
Macaca mulatta EILDTAGTEQFASMRDLYIKNGQGFILVYSLVNQQSFQDIKPMRDQIIRVKRY
Mus musculus EILDTAGTEQFASMRDLYIKNGQGFILVYSLVNQQSFQDIKPMRDQIIRVKRY
Pan troglodytes EILDTAGTEQFASMRDLYIKNGQGFILVYSLVNQQSFQDIKPMRDQIIRVKRY
Pteropus alecto EILDTAGTEQFASMRDLYIKNGQGFILVYSLVNQQSFQDIKPMRDQIIRVKRY
Rattus norvegicus EILDTAGTEQFASMRDLYIKNGQGFILVYSLVNQQSFQDIKPMRDQIIRVKRY
Bos taurus EILDTAGTEQFASMRDLYIKNGQGFILVYSLVNQQSFQDIKPMRDQIIRVKRY
Xenopus tropicalis EILDTAGTEQFASMRDLYIKNGQGFILVYSLVNQQSFQDIKPMRDQIIRVKRY
110 120 130 140 150
Homo sapiens ERVPMILVGNKVDLEGEREVSYGEGKALAEEWSCPFMETSAKNKASVDELFAE
Castor canadensis ERVPMILVGNKVDLEGEREVSYGEGKALAEEWSCPFMETSAKNKASVDELFAE
Macaca mulatta ERVPMILVGNKVDLEGEREVSYGEGKALAEEWSCPFMETSAKNKASVDELFAE
Mus musculus ERVPMILVGNKVDLEGEREVSYGEGKALAEEWSCPFMETSAKNKASVDELFAE
Pan troglodytes ERVPMILVGNKVDLEGEREVSYGEGKALAEEWSCPFMETSAKNKASVDELFAE
Pteropus alecto ERVPMILVGNKVDLEGEREVSYGEGKALAEEWSCPFMETSAKNKASVDELFAE
Rattus norvegicus ERVPMILVGNKVDLEGEREVSYGEGKALAEEWSCPFMETSAKNKASVDELFAE
Bos taurus ERVPMILVGNKVDLEGEREVSFGEGKALAEEWSCPFMETSAKNKASVDELFAE
Xenopus tropicalis EKVPMILVGNKVDLEGEREVSYGEGKALADEWNCPFMETSAKHKGSVDELFAE
160 170 183
Homo sapiens IVRQMNYAAQPNGDEGCCSACVIL
Castor canadensis IVRQMNYAAQPNGDEGCCSACVIL
Macaca mulatta IVRQMNYAAQPNGDEGCCSACVIL
Mus musculus IVRQMNYAAQPNGDEGCCSACVIL
Pan troglodytes IVRQMNYAAQPNGDEGCCSACVIL
Pteropus alecto IVRQMNYAAQPNGDEGCCSACVIL
Rattus norvegicus IVRQMNYAAQPNGDEGCCSACVIL
Bos taurus IVRQMNYAAQPNGDEGCCSACVIL
Xenopus tropicalis IVRQMNYASQPNGDDRCCSCVIL-
