# Supplementary material for: Rap2B drives tumorigenesis and progression of colorectal cancer through intestinal cytoskeleton remodeling
Source: Cell Death Dis. 2025 Apr 13;16(1):290. doi: 10.1038/s41419-025-07627-8 (PMC11994759; doi:10.1038/s41419-025-07627-8)
Supplement: Supplementary file 5 — Suppl Table 1 [file 41419_2025_7627_MOESM5_ESM.pdf]

Supplementary Table 1: list of real-time qPCR primers sets.

Gene	Sense primer	Antisense primer
Mouse		
Actin	5’-CAACGAGCGGTTCCGATGC-3’	5’-GCTGTCGCCTTCACCGTTC-3’
Rap2B	5’-CTCACCGTGCAGTTCGTAAC-3’	5’-CGATCTCTGCGAATAGCTCATCC-3’
plectin	5’-GCGGAGGAACAGTTGCAGAA-3’	5’-GCCCCTTGTACTCATTCAGTTG-3’
Human		
Actin	5’-AGAAAATCTGGCACCACACC-3’	5’-CTCCTTAATGTCACGCACGA-3’
Rap2B	5’-CTCACCGTGCAGTTCGTGAC-3’	5’-GGCAAATAGCTCGTCTACCGAG-3’
plectin	5’-AGCGTGAGAAGGAGAAGCTCCA-3’	5’-AGAGAGGAAGCTTTGCTGCAG-3’
