# Supplementary material for: Rap2B drives tumorigenesis and progression of colorectal cancer through intestinal cytoskeleton remodeling
Source: Cell Death Dis. 2025 Apr 13;16(1):290. doi: 10.1038/s41419-025-07627-8 (PMC11994759; doi:10.1038/s41419-025-07627-8)
Supplement: Supplementary file 6 — Full and uncropped western blots [file 41419_2025_7627_MOESM6_ESM.pptx]

## Slide 1
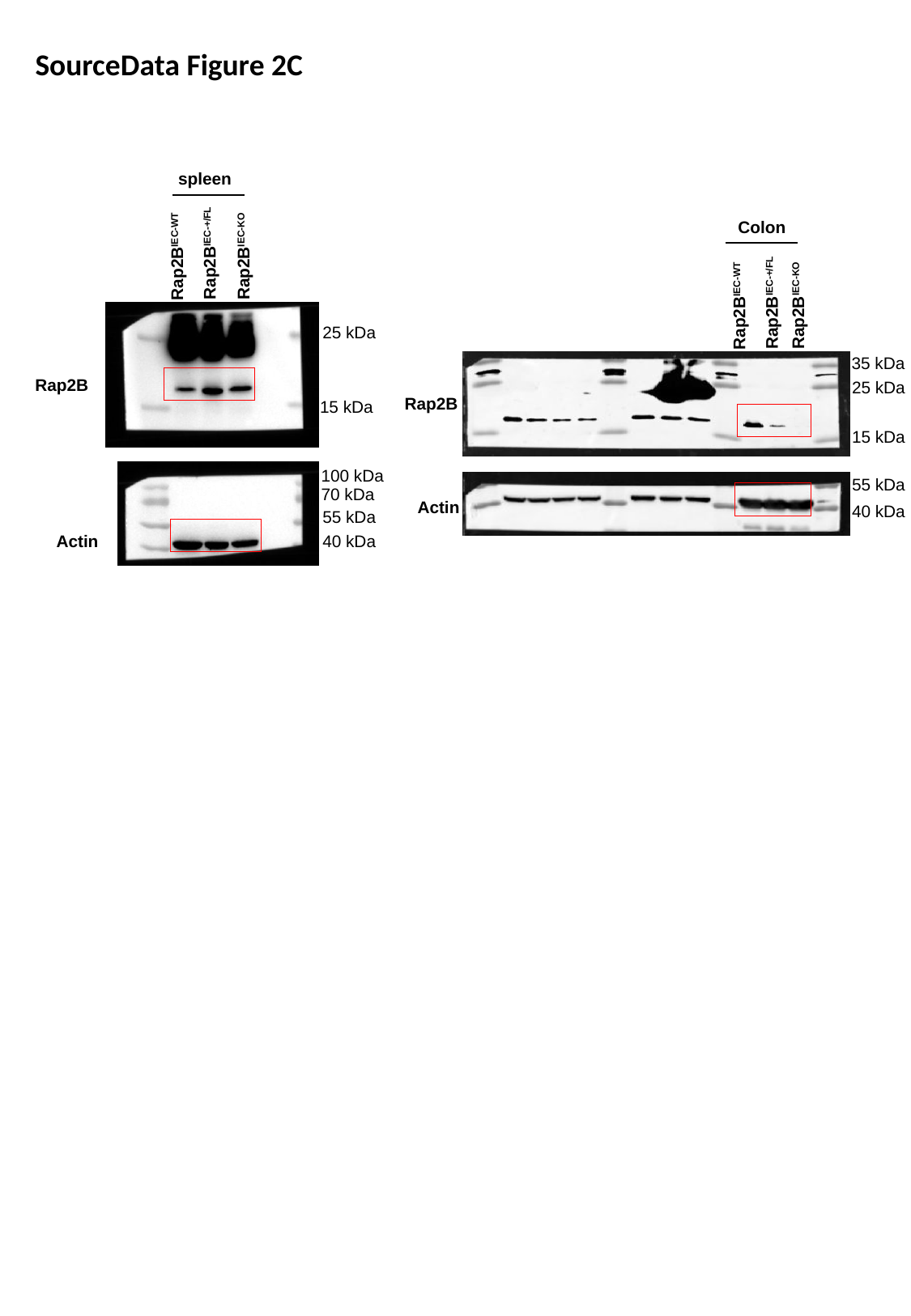

SourceData Figure 2C
spleen
Colon
Rap2BIEC-+/FL
Rap2BIEC-KO
Rap2BIEC-WT
Rap2BIEC-KO
Rap2BIEC-+/FL
Rap2BIEC-WT
25 kDa
35 kDa
Rap2B
25 kDa
Rap2B
15 kDa
15 kDa
100 kDa
55 kDa
70 kDa
Actin
40 kDa
55 kDa
Actin
40 kDa

## Slide 2
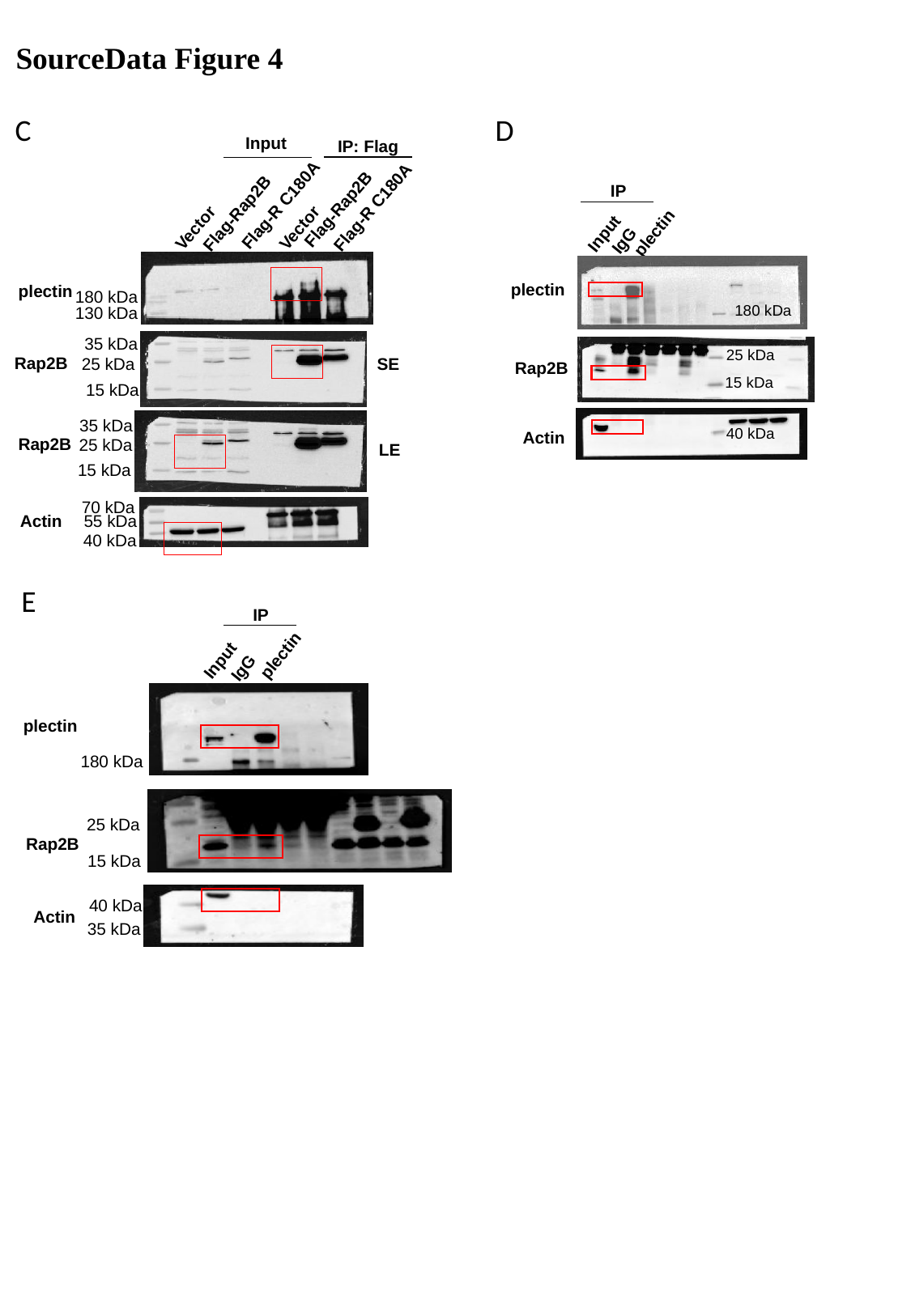

SourceData Figure 4
C
D
Input
IP: Flag
Flag-R C180A
Flag-R C180A
Flag-Rap2B
Flag-Rap2B
Vector
Vector
plectin
180 kDa
130 kDa
35 kDa
Rap2B
25 kDa
SE
15 kDa
35 kDa
Rap2B
25 kDa
LE
15 kDa
70 kDa
Actin
55 kDa
40 kDa
IP
plectin
Input
IgG
plectin
180 kDa
25 kDa
Rap2B
15 kDa
40 kDa
Actin
E
IP
plectin
Input
IgG
plectin
180 kDa
25 kDa
Rap2B
15 kDa
40 kDa
Actin
35 kDa

## Slide 3
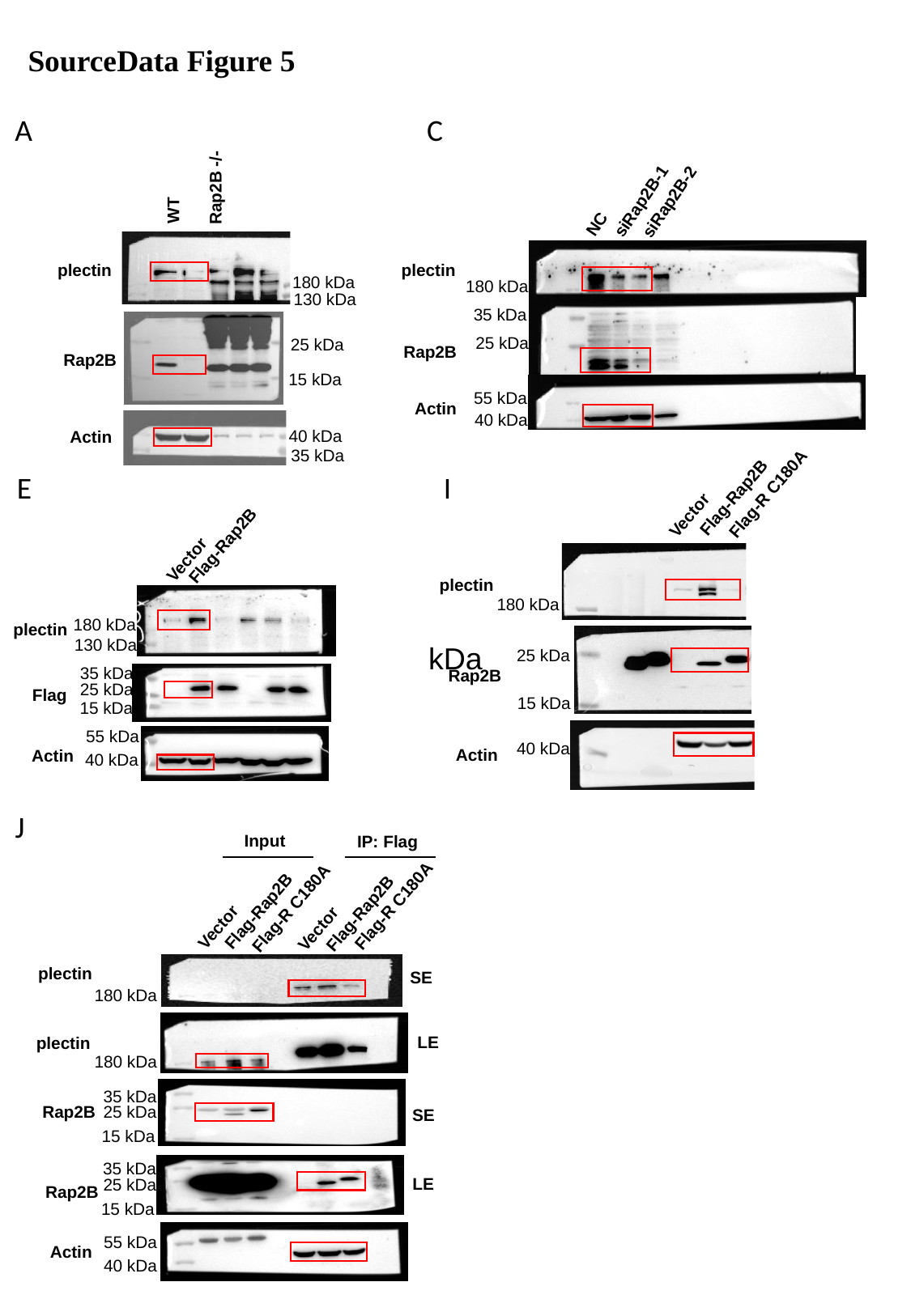

SourceData Figure 5
NC
siRap2B-1
siRap2B-2
plectin
Rap2B
Actin
180 kDa
35 kDa
25 kDa
55 kDa
40 kDa
A
C
Rap2B -/-
WT
plectin
180 kDa
130 kDa
25 kDa
Rap2B
15 kDa
40 kDa
Actin
35 kDa
E
I
Flag-R C180A
Flag-Rap2B
Flag-Rap2B
Vector
180 kDa
plectin
130 kDa
35 kDa
25 kDa
Flag
15 kDa
55 kDa
Actin
40 kDa
Vector
plectin
180 kDa
kDa
25 kDa
Rap2B
15 kDa
40 kDa
Actin
J
Input
IP: Flag
Flag-R C180A
Flag-R C180A
Flag-Rap2B
Flag-Rap2B
Vector
Vector
plectin
SE
180 kDa
LE
plectin
180 kDa
35 kDa
Rap2B
25 kDa
SE
15 kDa
35 kDa
LE
25 kDa
Rap2B
15 kDa
55 kDa
Actin
40 kDa

## Slide 4
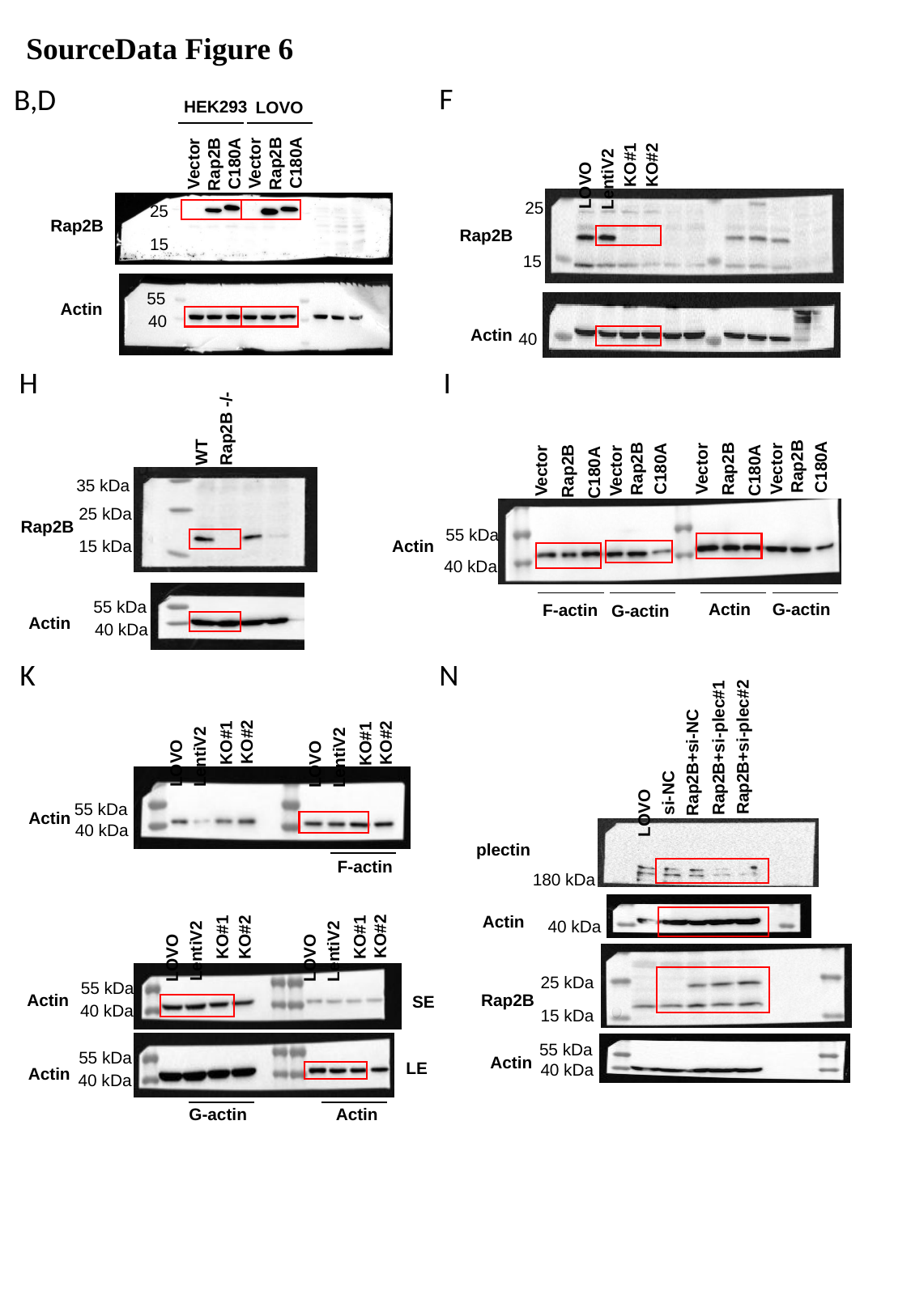

SourceData Figure 6
F
B,D
KO#2
LentiV2
LOVO
Rap2B
Actin
KO#1
HEK293
LOVO
Vector
Vector
Rap2B
C180A
Rap2B
C180A
Rap2B
Actin
25
25
15
15
55
40
40
H
I
Rap2B -/-
WT
Rap2B
Actin
Rap2B
Vector
Vector
Rap2B
Rap2B
Vector
Vector
Rap2B
C180A
C180A
C180A
C180A
Actin
Actin
G-actin
F-actin
G-actin
35 kDa
25 kDa
55 kDa
15 kDa
40 kDa
55 kDa
40 kDa
Rap2B+si-plec#2
Rap2B+si-NC
si-NC
LOVO
plectin
Actin
Rap2B
Actin
Rap2B+si-plec#1
K
N
KO#1
KO#1
KO#2
LentiV2
KO#2
LentiV2
LOVO
LOVO
Actin
F-actin
KO#1
KO#1
KO#2
LentiV2
LentiV2
KO#2
LOVO
LOVO
Actin
SE
LE
Actin
G-actin
Actin
55 kDa
40 kDa
180 kDa
40 kDa
25 kDa
55 kDa
40 kDa
15 kDa
55 kDa
55 kDa
40 kDa
40 kDa

## Slide 5
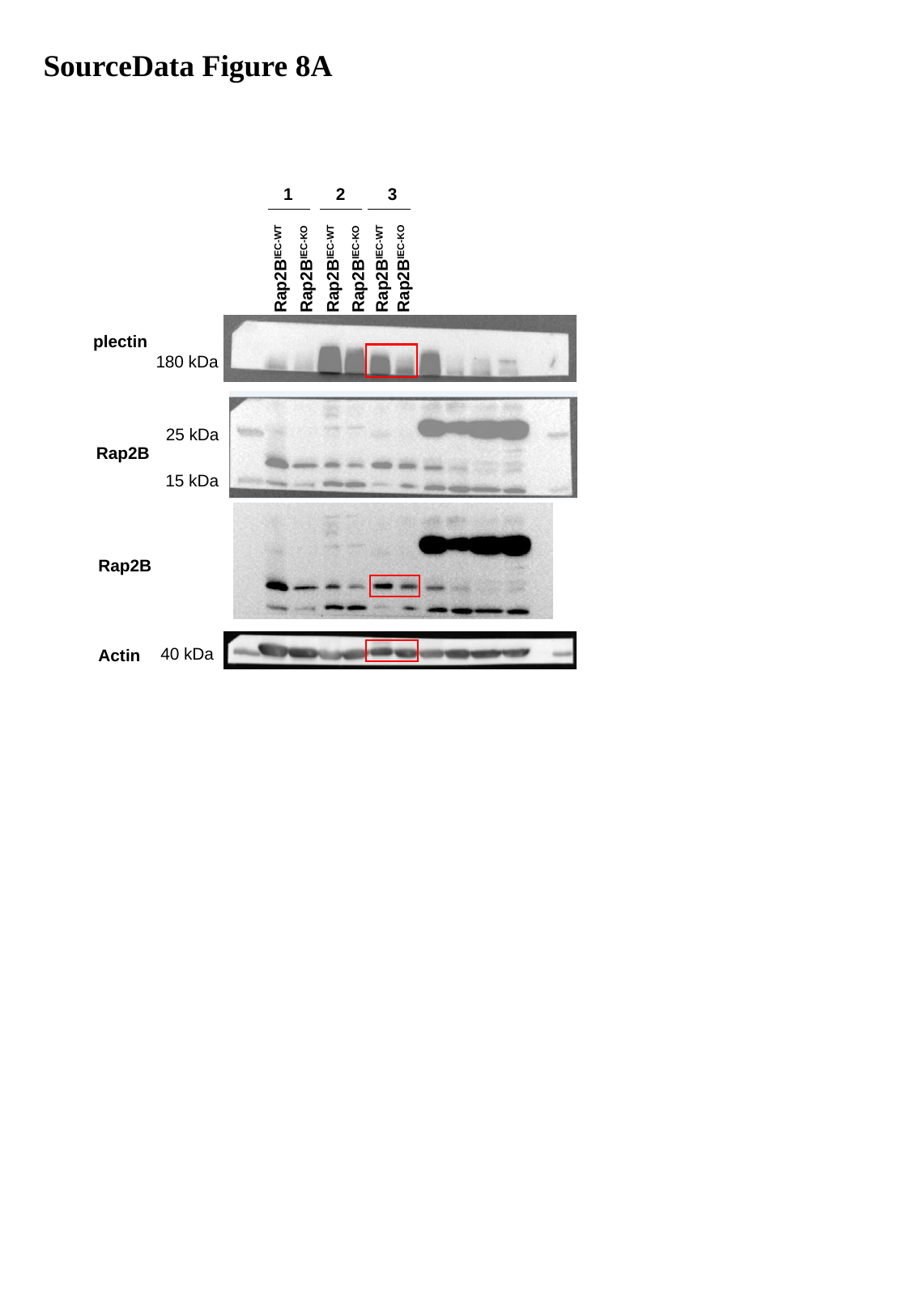

SourceData Figure 8A
1
2
3
Rap2BIEC-KO
Rap2BIEC-WT
Rap2BIEC-KO
Rap2BIEC-WT
Rap2BIEC-WT
Rap2BIEC-KO
plectin
180 kDa
25 kDa
Rap2B
15 kDa
Rap2B
40 kDa
Actin
